# Nebulized ivermectin for COVID-19 and other respiratory diseases, a proof of concept, dose-ranging study in rats

**DOI:** 10.1038/s41598-020-74084-y

**Published:** 2020-10-13

**Authors:** Carlos Chaccour, Gloria Abizanda, Ángel Irigoyen-Barrio, Aina Casellas, Azucena Aldaz, Fernando Martínez-Galán, Felix Hammann, Ana Gloria Gil

**Affiliations:** 1 ISGlobal, Hospital Clínic - Universitat de Barcelona, Rosello 132, 5ª 2ª, 08036 Barcelona, Spain; 2 Ifakara Health Institute, 67501 Ifakara, United Republic of Tanzania; 3 Facultad de Medicina, Universidad de Navarra, 31008 Pamplona, Spain; 4 Centro de Investigación Médica Aplicada, 31008 Pamplona, Spain; 5 Clínica Universidad de Navarra, 31008 Pamplona, Spain; 6 Facultad de Farmacia y Nutrición, Universidad de Navarra, 31008 Pamplona, Spain; 7 Drug Development Unit Universidad de Navarra, 31008 Pamplona, Spain; 8 Departament de Fonaments Clínics, Facultat de Medicina, Universitat de Barcelona, Barcelona, Spain; 9 Department of General Internal Medicine, Clinical Pharmacology and Toxicology, Inselspital, Bern, University Hospital, 3010 Bern, Switzerland

**Keywords:** Drug delivery, Toxicology, Drug development, Preclinical research

## Abstract

Ivermectin is a widely used antiparasitic drug with known efficacy against several single-strain RNA viruses. Recent data shows significant reduction of SARS-CoV-2 replication in vitro by ivermectin concentrations not achievable with safe doses orally. Inhaled therapy has been used with success for other antiparasitics. An ethanol-based ivermectin formulation was administered once to 14 rats using a nebulizer capable of delivering particles with alveolar deposition. Rats were randomly assigned into three target dosing groups, lower dose (80–90 mg/kg), higher dose (110–140 mg/kg) or ethanol vehicle only. A toxicology profile including behavioral and weight monitoring, full blood count, biochemistry, necropsy and histological examination of the lungs was conducted. The pharmacokinetic profile of ivermectin in plasma and lungs was determined in all animals. There were no relevant changes in behavior or body weight. There was a delayed elevation in muscle enzymes compatible with rhabdomyolysis, that was also seen in the control group and has been attributed to the ethanol dose which was up to 11 g/kg in some animals. There were no histological anomalies in the lungs of any rat. Male animals received a higher ivermectin dose adjusted by adipose weight and reached higher plasma concentrations than females in the same dosing group (mean C_max_ 86.2 ng/ml vs. 26.2 ng/ml in the lower dose group and 152 ng/ml vs. 51.8 ng/ml in the higher dose group). All subjects had detectable ivermectin concentrations in the lungs at seven days post intervention, up to 524.3 ng/g for high-dose male and 27.3 ng/g for low-dose females. nebulized ivermectin can reach pharmacodynamic concentrations in the lung tissue of rats, additional experiments are required to assess the safety of this formulation in larger animals.

## Introduction

As of August 19, 2020, there have been more than 22 million COVID-19 cases causing over 785,000 deaths worldwide. In the absence of a vaccine, numerous efforts are ongoing to develop drug-based strategies to prevent, treat or reduce the transmission of the virus. Data on several drug regimens suggest lack of efficacy for lopinavir-ritonavir^1^, hydroxychloroquine as prophylaxis^2^ or even harmfulness such as high-dose hydroxychloroquine for prophylaxis^3^ while remdesivir^4^ and dexamethasone^5^ seem to improve patients’ outcome.

Ivermectin is a widely used antiparasitic drug with known efficacy against several single-strain RNA viruses including Dengue^6^, Zika^7^ and other viruses^8^. The effect on flaviviruses could be explained by a reduction of the viral penetration into the nucleus via an effect on the host´s importin alpha/beta1^9^, inhibition of the viral helicase^8^ or yet to be described mechanisms.

Caly et al. showed a significant reduction of SARS-CoV-2 replication after incubating Vero cells, a cell line derived from African Green Monkey kidney epithelial cells, for 48 h with ivermectin concentrations not readily attainable in vivo^10^. Yet, these findings stirred widespread interest and together with a preprint report of potential benefit of a single dose of ivermectin 150 ug/kg on in-hospital mortality has resulted in extensive use of ivermectin for the treatment or prevention of COVID-19, particularly in Latin-America. There are currently 27 clinical trials registered in clinicaltrials.gov testing ivermectin alone or in combination against COVID-19.

Despite of the mismatch between ivermectin´s pharmacokinetics and the IC_50_ reported by Caly et al., the drug may still prove useful for COVID-19 patients given marginal in vivo effects against dengue in spite of even higher estimated in vitro IC_50_ values (> 10 μM) in Vero cells^6,11^, known anti-inflammatory effects^12,13^ and potential additional immunomodulation through an interaction with nicotinic receptors^14^. Another proposed mechanism is the increase of the airway epithelial cell expression of the angiotensin-converting enzyme II (ACE-2) viral entry receptor mediated by activation of nicotinic acetylcholine receptors (nAChR)^15^, primarily the α7 subtype. This would help explain the additional susceptibility of smokers to Covid-19. Inhibition of α7-nAChR may suppress this process, thereby reducing within-host infectivity and transmission. Krause et al. found a comparatively low α7-nAChR IC_50_ for ivermectin of 0.156 μM, a concentration realistically attainable even on oral drug dosing^16^.

Inhaled therapy could be used to instantaneously reach and maintain effective concentrations in lung tissue. Other authors have previously described successful delivery of antiparasitic drugs using nebulizers that create a particle size capable of reaching alveoli in hospital and community settings^17,18^. We are only aware of a single previous study using nebulized ivermectin, Ji et al.^19^ exposed Sprague–Dawley rats to a known concentration of ivermectin in chamber air and determined the inhaled LD_50_ at 4 h to be 3690 (2710–5010) mg/m^3^ and the no adverse events were seen in rats exposed to a concentration of 380 mg/m^3^ for 21 consecutive days. The study design of Ji et al., however seems intended as an environmental toxicity study, not to guide a therapeutic use of the drug.

Here we report of a pilot rat-model evaluating the feasibility of therapeutic delivery of nebulized ivermectin. The main objectives were to assess the ivermectin pharmacokinetics in plasma and lung tissue as well as safety of this formulation through a comprehensive toxicology profile.

## Methods

### Formulation

We unsuccessfully attempted to produce a water soluble-solution by combining surfactants such as polysorbate or co-solvents such as propylene glycol or glycerin. Given solubility issues and that ethanol does not interfere with ivermectin pharmacokinetics^20^, ivermectin powder API was diluted in pure ethanol to reach three different concentrations, 7 mg/ml, 10 mg/ml and 14 mg/ml and kept in opaque flasks at 5 °C. Stability tests using HPLC conducted one week after dilution showed no changes in the original concentrations. All solutions were used within one week of preparation.

### Rats

We used adult, 12-weeks old, Sprague–Dawley rats that were kept in groups of 2–3 subjects of the same sex per cage at the animal research facilities of the University of Navarra. Light, temperature, humidity and feeding conditions followed the local standard operating procedures. Rats in the intervention groups were identified as TM/TF (males/females) plus a correlative number 1–6. Rats in the control group were identified as VCM or VCF for male and female respectively. Cages were identified with the study name and each rat was marked with its individual code in the base of the tail using a non-toxic permanent marker.

### Procedures

**Figure 1:**
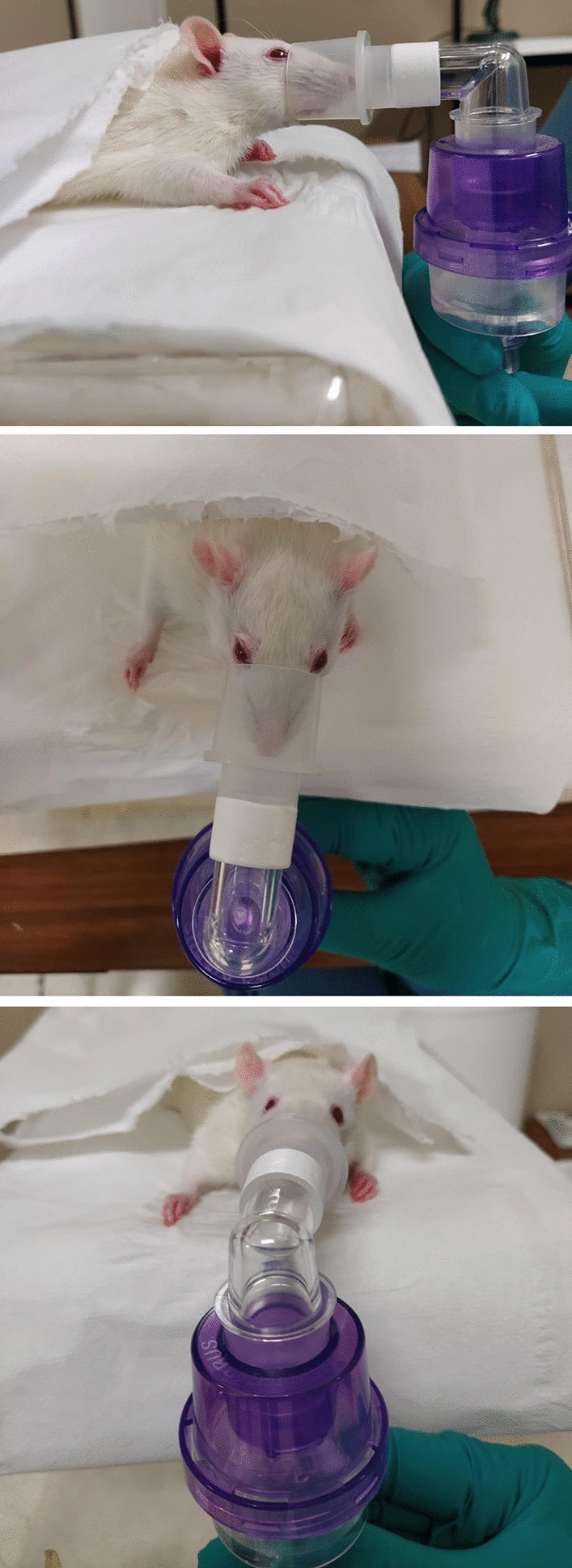
Nebulization procedure.

For the nebulization, we used a Micro Cirrus device (Intersurgical, Berkshire, UK) with a driving oxygen flow of 8 L/min which can deliver aerosol particles of 0.5–2 μm in diameter resulting in alveolar deposition. A volume of 3 ml of the solutions was used. A 22-mm elbow piece that covered the rats’ nose and mouth was used to deliver the cloud (Fig. 1). The oxygen flow was kept until the reservoir was empty at visual inspection for an average nebulizing time of 9 min. The reservoirs were weighed before and after the nebulization to confirm the final weight was the same as the empty one, before the procedure.

The oral lethal concentration 50% (LC_50_) for rats has been estimated by Merck in 42.8–52.8 mg/kg for males and 44.3–52.8 mg/kg for females^21^. The rats for this study were randomly assigned into three groups: (a) lower dose, aiming at twice the lower boundary of the LC_50_ for their sex; (b) higher dose, aiming at twice the upper boundary of the LC_50_ for their sex and (c) controls receiving only the ethanol vehicle. The sample was chosen according to usual practice in dose-range finding pilots. Treatment groups included three males and three females each, rats from each sex were housed in separate cages. The first three males received the lower dose, the first three females received the lower dose, the remaining three in each cage were assigned to the missing dosing group and the last rat in each cage remained untreated.

The intervention was administered in dose-groups under anesthesia with ketamine and diazepam 75/5 mg/kg, intraperitoneal, single dose. Post-administration, the rats were monitored until recovery for 60–90 min on a thermic blanket and then monitored daily using a modified Irwin test^22^ until euthanasia. The weight of all subjects was recorded at baseline, day 3 and at the day of euthanasia. Additionally, a full blood count and a biochemistry toxicology panel including total serum proteins, albumin, aspartate aminotransferase (AST), alanine aminotransferase (ALT), bilirubin, total cholesterol, glucose, creatinine, urea, creatine phosphokinase (CPK)and lactate dehydrogenase (LDH) was performed in one subject per sex/group at euthanasia.

Plasma samples for determination of ivermectin levels were obtained at 1, 4 and 24 h after administration and at the time of euthanasia (days 3, 5 or 7). Blood samples of 0.5 ml were obtained under brief induction anesthesia with inhaled isoflurane 1.5–2% from the retro orbital plexus, centrifuged and the plasma frozen at − 80 °C until processing.

**Figure 2:**
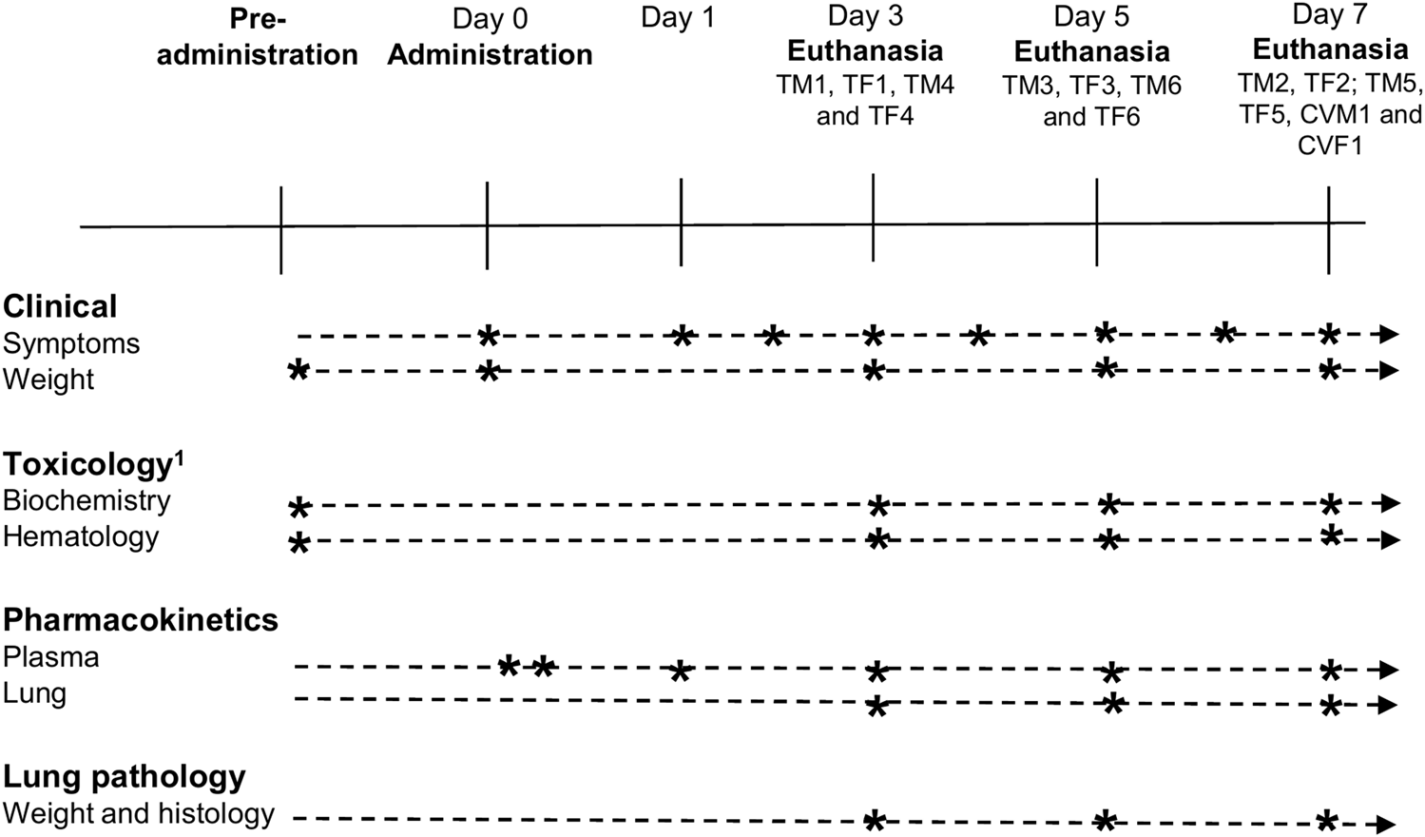
Summary and timeline of study procedures. TM#/TF#, CVM and CVF. ^1^One animal per sex and treatment group.

A t the end of their corresponding study period (Fig. 2), subjects were euthanized using a CO_2_ chamber. A macroscopic necropsy was performed in all subjects including external and in situ examination of all organs. The lungs and livers were extracted, measured and weighted separately. One lung and one liver sample from every subject was preserved in formaldehyde and sent to Patconsult LAB (Barcelona, Spain) for histopathological examination.

### Ivermectin levels

Ivermectin plasma levels were determined using a variation of a previously described HPLC-FLD^23^ with a detection limit of 0.1 ng/ml.

Lung tissue (0.5 mg) was homogenized in 1 ml of acetonitrile using an Ultra-turrax homogenizer and the sonicated for 15 min, 100 mcl of the homogenized lung were processed in the HPLC-FLD using the plasma methods^24^.

### Calculations, data analysis and software

Given that ivermectin deposits in adipose tissue^25^ and that differences in ivermectin’s PK based on body composition have been previously described^26^, the dose per gram of body fat was calculated in all rats. The adipose weight of the rats according to sex was estimated using the formula described by Ferrel and Koong^27^. Comparison of baseline characteristics was done using the Wilcoxon Rank Sum test in Stata 16 Software (StataCorp. 2019. Stata Statistical Software: Release 16. College Station, TX: StataCorp LLC). Body weigh-PK by sex graphs were done in Microsoft Excel (Microsoft corporation, 2018).

Data set checkout and visualizations of pharmacokinetic data were performed in GNU R (R Core Team [2020] R: A language and environment for statistical computing, version 3.6.3, R Foundation for Statistical Computing, Vienna, Austria, https://www.R-project.org/). Non-compartmental analysis (NCA) was performed using Pkanalix (Monolix version 2019R2. Antony, France: Lixoft SAS, 2019. https://lixoft.com/products/monolix/). Peak plasma concentration (${C}_{max}$), and time to peak plasma concentration (${T}_{max}$) were read directly from the profiles. Terminal elimination half-life (${t}_{1/2}$) was calculated as ${t}_{1/2}=ln(2)/{\lambda }_{z}$ after estimating ${\lambda }_{z}$ from the final two measurements of each profile. The area under the plasma concentration–time curve (AUC) from 0 to 168 h post-dose ($AU{C}_{0-168h}$) was calculated using the linear up/log down method. Mean plasma concentrations from 0 to 168 h post-dose are given as ${C}_{avg.,0-168h}$. Lung tissue concentrations were back-calculated from lung densities reported by El-Khatib et al. in Sprague–Dawley female rats aged 15–90 weeks^28^. At 15 weeks of age, the average lung density was about 0.4 g/cm^3^, and we therefore converted 1 g of lung tissue to a volume of 2.2 ml.

### Ethics

All procedures were reviewed and approved by the Animal research ethics committee of the University of Navarra (Approval Number E11/20 from CEEA 002/20).

### Regulatory compliance

All methods were carried out in accordance with relevant guidelines and regulations.

## Results

### Baseline characteristics

The baseline characteristics of the rats in by sex is presented in Table 1. Male rats had an approximate median weight 100 g (50%) higher than females (*p*-value = 0.002) and different body composition resulted in females having three times the estimated adipose weight (median 92.9 vs. 29.1 g, *p*-value = 0.002).

**Table 1:** Baseline characteristics of the rats in each group.

Variable	Sex		Total
	Male	Female	
Baseline weight (g)^a^	346.9 (331.5–372.5) [7]	237 (218.8–249.5) [7]	277.55 (237–346.9) [14]
Fat weight (g)^a^	29.1 (27.5–31.8) [7]	92.9 (81.6–101.1) [7]	55.3 (29.1–92.9) [14]
**Dosage**			
Dose (mg)^b^			
None	1 (14%)	1 (14%)	2 (14%)
Lower dose	3 (43%)	3 (43%)	6 (43%)
Higher dose	3 (43%)	3 (43%)	6 (43%)
Dose (mg/Kg)^a^	98.7 (86.5–112.8) [7]	96.0 (83.6–132.5) [7]	97.3 (86.5–116.5) [14]
Dose per gram of fat^a^	1.2 (1.0–1.3) [7]	0.3 (0.2–0.3) [7]	0.4 (0.2–1.2) [14]

^a^Median (IQR) [n]. ^b^n (Column percentage).

### Intervention and immediate recovery

The recommended minimal volume to generate nebula with the device used is of three ml, given that our formulation is ethanol/based, this volume set the minimal ethanol dose given with the intervention in 2.3 g (equivalent to 3 ml). All animals received the intervention uneventfully and recovered from anesthesia within 90 min. At the time of the first blood sample (1 h after the intervention), they showed slight instability and lethargy which was attributed to the alcohol dose and had recovered fully by the time of the second blood sample, 4 h after the intervention.

### Ivermectin dose

The animals in the lower dose group received a median dose of 89 mg/kg of ivermectin, ranging 86–98 mg/kg for males and 88–95 mg/kg for females which correlates well with the target of twice the lower boundary of the oral LD_50_. The animals in the higher dose group received a median dose of 121 mg/kg of ivermectin, ranging 108–116 mg/kg for males and 126–141 mg/kg for females which represents 2.0 to 2.2-fold the upper boundary of the oral LD_50_ for males and 2.3 to 2.6-fold the upper boundary of the LD_50_ for females. However, the significantly different adipose weight resulted in male rats receiving much higher doses of ivermectin per gram of fat than females (3.2 to fourfold in the higher dose group, and 3.9 to 5.7-fold in the lower dose group), weights and doses of all animals are presented in Table 2.

**Table 2:** Weight, body composition, doses, plasma and lung levels of all rats.

Group	Subject	Baseline weight (g)	Fat weight (g)^a^	Body fat (%)	Total dose (mg)	Dose (mg/kg)	Dose per gram of fat (mg/g)	Plasma levels 1 h (ng/ml)	Plasma levels 4 h (ng/ml)	Plasma levels 24 h (ng/ml)	Plasma levels 72 h (ng/ml)	Plasma levels 140 h (ng/ml)	Plasma levels 168 h (ng/ml)	Lung levels (ng/g) (time)
Lower dose males	TM1	303.9	24.69	8	30	98.7	1.2	55.0	29.1	81.8	74.6	–	–	75.6 [72 h]
	TM2	333.2	27.71	8	30	90.0	1.1	26.1	29.8	96.7	–	–	25.9	110.6 [168 h]
	TM3	346.9	29.12	8	30	86.5	1.0	36.4	48.3	80.0	–	38.2	–	141.2 [140 h]
Higher dose males	TM4	386.9	33.24	9	42	108.6	1.3	35.9	44.2	142.9	63.4	–	–	592.9 [72 h]
	TM5	360.6	30.53	8	42	116.5	1.4	52.5	39.6	186.7	–	–	56.0	524.3 [168 h]
	TM6	372.5	31.76	9	42	112.8	1.3	52.4	39.9	126.4	–	58.5	–	446.8 [140 h]
Higher dose females	TF1	211.6	77.35	37	30	141.8	0.4	9.8	9.9	33.6	15.0	–	–	339.2 [72 h]
	TF2	226.4	86.24	38	30	132.5	0.3	63.4	10.9	22.8	–	–	13.2	94.7 [168 h]
	TF3	237	92.91	39	30	126.6	0.3	11.2	8.4	58.6	–	13.4	–	170.6 [140 h]
Lower dose females	TF4	218.8	81.61	37	21	96.0	0.3	11.4	10.5	27.4	6.6	–	–	250 [72 h]
	TF5	251.2	102.23	41	21	83.6	0.2	40.9	8.1	20.7	–	–	6.7	27.3 [168 h]
	TF6	238.4	93.81	39	21	88.1	0.2	9.8	9.5	10.3	–	6.9	–	13.8 [140 h]
Control (m)	CVM1	331.5	27.54	8	0	**	**	**	**	**	**	**	**	** [168 h]
Control (f)	CVF1	249.5	101.09	41	0	**	**	**	**	**	**	**	**	** [168 h]

**Below quantification limit.

^a^Ferrel and Koong method.

All rats received 3 ml (2.3 g) of inhaled ethanol, which resulted in weight-adjusted ethanol doses of 6.1–7.7 g/kg for males (Table S18) and 9.4–11.1 g/kg for females (Table S19) which is lower than or overlapping with the oral ethanol LD_50_ described for rats of a similar age (10.0–11.2 g/kg)^29^.

### Behavior, symptoms and weight

All animals had a normal behavior and central nervous system assessment throughout the study according to the modified Irwin procedure (Tables S3-S17).

All animals had a normal weight for their age at baseline, except for one male (TM1) who was 17 g (5%) below the lower limit of the normality for 12 weeks (321 g).

All males except one in the lower dose group had lost weight at day three post intervention, ranging from 2 to 4.2% of their baseline weight. The control male, nebulized with ethanol vehicle only, lost 3.5% of its baseline weight by day three. All males except one in the higher dose group that lost further 0.6%, had started to gain weight or recovered their baseline value at the time of euthanasia (Table S18). All females except two (one in the lower dose group and one in the higher dose group) had lost weight at day three post intervention, ranging from 0.4 to 3.4% of their baseline weight. The control female lost 0.4% of its baseline weight by day three. All females had started to gain weight or recovered their baseline value at the time of euthanasia (Table S19).

### Toxicology labs

Male rats had no significant hematological changes regardless of ivermectin dose group or ethanol dose adjusted by weight (Table S20). All but two female rats had a slight anemia with hemoglobin levels 0.1 to 1.7 g/dL below the minimum normal value. Additionally, all females had total red blood cells below the normal limit (median 0.8 × 10^6^/ul) and slightly increased mean corpuscular values (median 3.5 fl above the upper normal limit) (Table S21).

Animals in both groups presented a delayed increase (two to threefold the upper level of normality) in creatine kinase (CPK) and lactate dehydrogenase (LDH), this effect was seen earlier in females (all euthanized after 140 h post administration) than in males (only those euthanized at 168 h). Except for one female in the higher ivermectin dose which had AST/ALT values 1.5-fold the upper limit, liver enzymes and bilirubin were normal or below the lower limit in all rats. Two males and all but one female had slightly increased creatinine values. All blood biochemistry results are presented in Supplementary Tables S1 and S2 for males and females respectively.

### Necropsy

One male in the higher dose group (TM6) had a patchy pattern in both lungs (Figure S1). There were no macroscopic findings in the thoracic or abdominal organs of any other subject. The absolute and relative-to-body weights of lungs and livers from all subjects where within normal values (Tables S22 and S23). There were no pathologic changes in the histology of liver and lungs samples examined, including the lung sample from the male rat with a macroscopic patchy pattern (Figure S2).

### Pharmacokinetics

**Figure 3:**
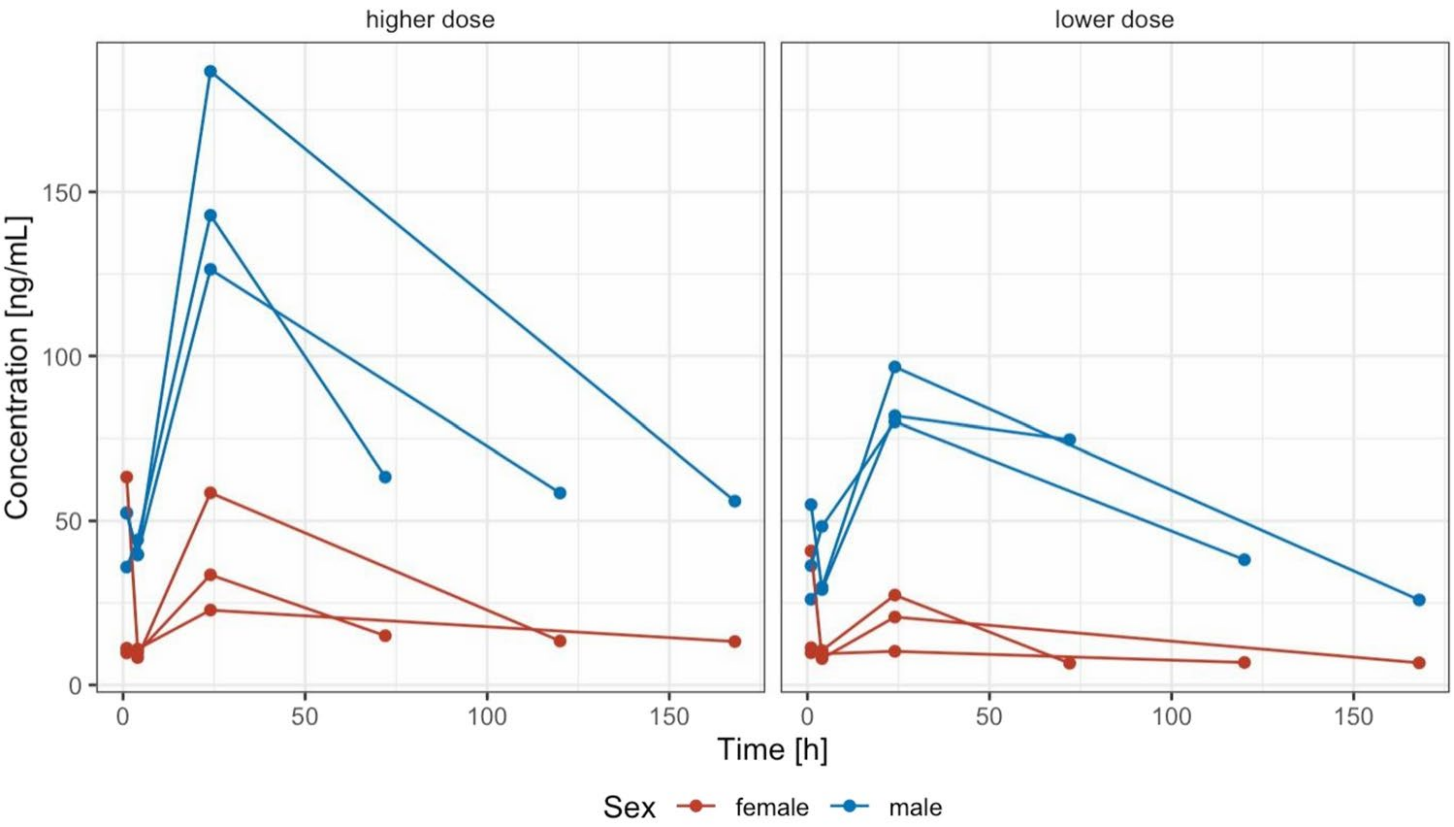
Individual plasma concentration–time curves stratified by sex and dose administered (lower dose = 84–98 mg/kg, higher dose 106–140 mg/kg).

**Figure 4:**
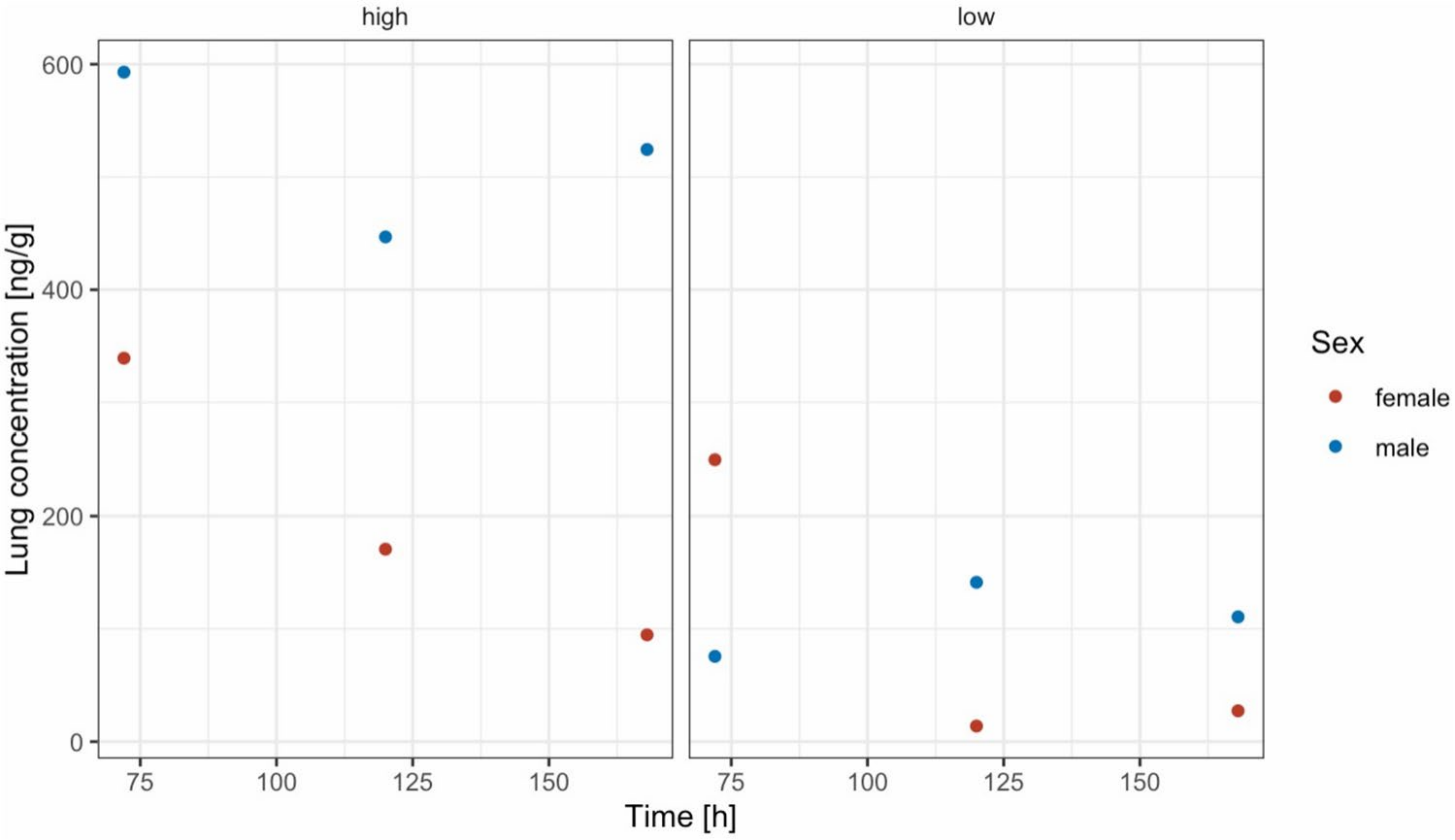
Lung concentrations at necropsy for all animals stratified by dose administered and sex (lower dose = 84–98 mg/kg, higher dose 106–140 mg/kg).

Individual plasma profiles by dose group and sex are shown in Fig. 3. Secondary pharmacokinetic parameters in plasma are given in Table 3. Concentrations in lung tissue remained detectable in all animals at time of necropsy (72–168 h, Table 2, Fig. 4).

**Table 3:** Secondary pharmacokinetic parameters derived from non-compartmental analysis of plasma concentration–time profiles.

Dose group	Sex	Units	Cmax	Tmax (a)	Clast	Cavg 0–168 h	t1/2	AUC 0–168 h
			(ng/mL)	(h)	(ng/mL)	(ng/mL)	(h)	(ng*h/mL)
Lower dose	Female (n = 3)	Mean (SEM)	26.2 (8.9)	24	6.7 (0.1)	4.7 (1)	127.5 (52.7)	1610.5 (275.1)
		Range	10.3–40.9	1–24	6.6–6.9	3.0–6.4	23.4–193.7	1330.3–2160.7
	Male (n = 3)	Mean (SEM)	86.2 (5.3)	24	46.2 (14.6)	17.3 (1.5)	175.7 (92.9)	9679.6 (958.9)
		Range	80.0–96.7	24–24	25.9–74.6	14.9–20.0	75.8–361.3	8387.0–11,552.8
Higher dose	Female (n = 3)	Mean (SEM)	51.8 (9.2)	24	13.9 (0.6)	7.5 (1.0)	370.8 (327.6)	3128.2 (526.2)
		Range	33.6–63.4	1–24	13.2–15.0	5.7–9.2	41.3–1026.0	2291.6–4099.3
	Male (n = 3)	Mean (SEM)	152.0 (18.0)	24	59.3 (2.2)	25.0 (1.5)	70.1 (14.6)	13,461.7 (2447.9)
		Range	126.4–186.7	24–24	56.0–63.4	23.1–27.9	40.9–86.4	9707.4–18,060.1

AUC 0–168 h, area under the plasma concentration–time curve from time zero to 168 h; C_avg_ 0–168 h, mean plasma concentration from 0 to 168 h, Clast, last observed plasma concentration, C_max_, maximum plasma concentration; t1/2, terminal plasma elimination half-life; SEM, standard error of the mean; T_max_, time to reach C_max_. (a) media.

**Figure 5:**
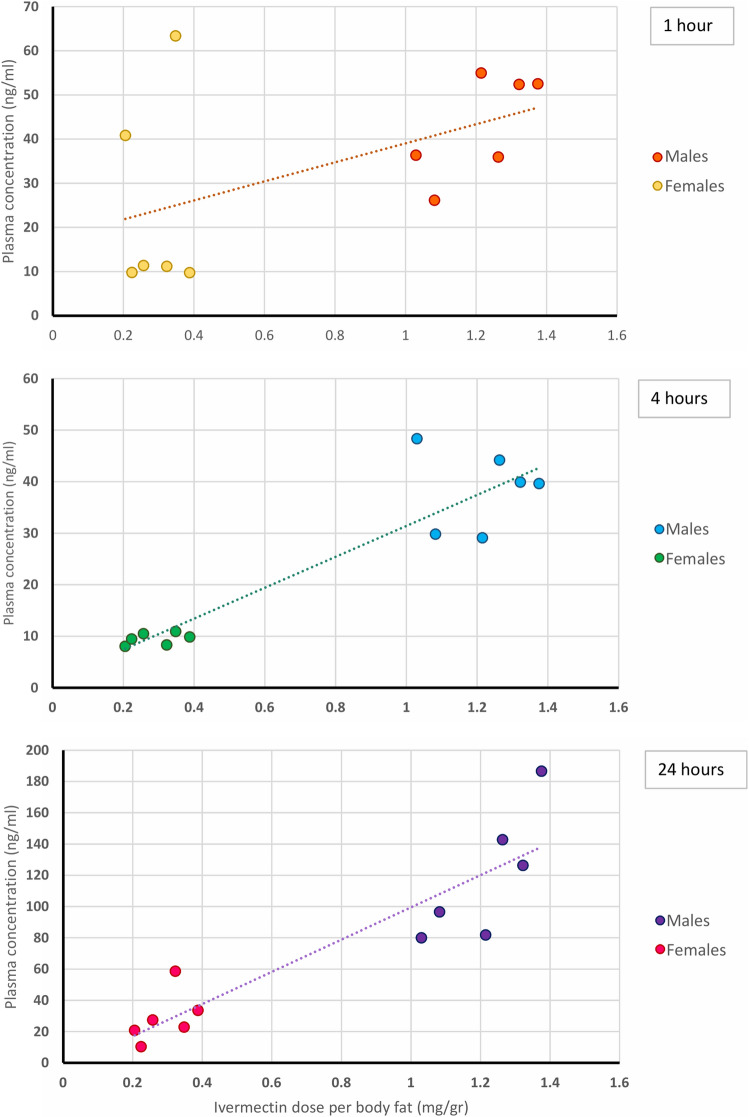
Relationship between dose per gram of fat and ivermectin plasma levels at 1, 4 and 24 h after nebulization in rats.

The relationship between the ivermectin dose per gram of fat and the plasma concentrations at 1, 4 and 24 h is shown in Fig. 5.

## Discussion

### Dose and body composition

Although the total and weight-adjusted ivermectin doses were comparable between males and females in both dose groups, the higher estimated adipose weight of female rats resulted in a 3 to fourfold dose of ivermectin per gram of fat. Ivermectin accumulates in adipose tissue^25^, this deep compartment affects systemic concentrations of the drug in subjects with different body composition and sex-based differences have been well described before^26,30^. In this experiment there was a direct relationship between the dose per gram of fat and the plasma levels reached at 1, 4 and 24 h. Nebulized ivermectin reached a C_max_ twice as high and had an AUC 4 to sixfold higher in male rats regardless of the dosing group. There was also a tendency for lung levels of male rats to be higher regardless of the dosing group.

### Toxicology

The hematological changes observed in most female rats, regardless of dosing group, (mild macrocytic anemia and reduced total RBC) are already present in animals euthanized 72 h after the intervention and could either precede the intervention or be the consequence of the proportionally larger blood loss due sampling in this group. The elevated CPK and LDH observed 5–7 days after the intervention seem to be of muscular origin given normal values of bilirubin and liver enzymes in all rats; we hypothesize these changes are in relationship with the high ethanol dose received (up to 11 g/kg of ethanol which is the equivalent of one standard alcohol unit per kg or weight) since they are also present in the control animals that did not receive any ivermectin. Non-traumatic rhabdomyolysis has been reported before in relationship with acute alcohol intoxication^31^ and this is compatible with the slight increase in creatinine values seen in most animals. Alcohol intoxication is also compatible with the described initial weight loss and posterior recovery. The anesthesia used during the intervention and for the PK sampling could have played a role in the CPK/LDH elevation but isoflurane is not known to increase LDH^32^ and ketamine-induced elevations are generally of smaller magnitude^33^. The ethanol dose that a 70-kg human adult would receive with a nebulized 3 ml solution would be 2.36 g (the equivalent of 1/5 of one standard alcohol unit) or 300-fold less than the dose received by the rats in this study. In any case, care must be taken as recreational use of inhaled ethanol can be used in binge-like patterns, even if immediate safety concerns are relatively minor^34^.

### Pharmacokinetics

Upon visual inspection most concentration–time curves show a biphasic profile. We attribute this to double absorption first through inhalation (fast uptake) and then oral absorption (slower uptake) of swallowed drug. It is notable that the second peak is higher than the first, indicating that the majority of drug was swallowed. This is a common phenomenon in nebulized vehicles where subjects are not skilled at inhalation or unable to comply with instructions. We therefore assume that considerably higher lung exposure could be achieved in intubated animals. Nebulized administration of ivermectin to human volunteers would presumable also allow for better deposition of the drug in the respiratory tract and lower circulating levels.

We attempted to characterize the pharmacokinetic behavior and fractions absorbed via both ways using a population-based approach. As the initial uptake in the lungs occurs very early in the profiles where sampling was not frequent, we were unable to construct robust models. Future trials could overcome this by more intensively sampling subjects during the first hours post-dose.

### Achieving pharmacodynamic targets

The highest observed plasma concentration was 186.7 ng/mL which corresponds to 0.21 μM/L and is clearly below the IC_50_ ~ 2 μM reported by Caly et al.^10^. Previous experience in Dengue virus infection with the translation of in vitro results to effectivity in human subjects has shown that Vero cell results should be interpreted qualitatively, not quantitatively. The actual IC_50_ in humans and even respiratory epithelial cells may be quite different.

Concentrations are, however, above the nicotinergic acetylcholine receptor (nAChR) IC_50_ for ivermectin as estimated by Krause et al.^16^. Lung tissue concentrations in male rats in the high-dose arm were well above this concentration after 72–168 h (Figure S3). Without a more thorough understanding of the absorption process, it is difficult to extrapolate what concentrations could be achieved with proper inhalation technique. It is, however, safe to say that ivermectin lung delivery with nebulized formulations can maintain detectable concentrations for 7 days.

The delivery method investigated here is not directly translatable to human clinical trials. Safety has to be demonstrated not only for ivermectin but also the ethanol vehicle. Our experience in man with ivermectin is with oral formulations only (sporadic case reports of compassionate use of veterinary parenteral products not considered). Oral bioavailability is not known, and it is conceivable that absorption via lung tissue is more complete as there is no first-pass effect compared to oral administration. We may therefore see higher, possibly toxic, systemic exposure on inhalation. Preliminary safety and dose-finding studies should be conducted with a second arm with oral dosing of the same amounts to quantify relative bioavailability.

Ivermectin has shown in vitro and in vivo effects against other flaviviridae. How well the respective modes of action translate to SARS-CoV-2 is under investigation. Until we have more appropriate in vitro results for key steps such as viral entry via ACE2 receptors or nicotinic receptors, or viral replication, drug regimen design will be difficult.

### Other potential disease targets

Ivermectin is efficacious against several helminths of human and veterinary importance that have a pulmonary phase during their lifecycle^35^. There are raising concerns about emerging anti-helminthic resistance and its potential impact on human health and livelihood^36^, targeting these parasites while they are in the lungs (some of them with extended lung residence periods) with much higher concentrations than usually available after oral administration provides a potential answer to these concerns.

In human medicine, this could impact individual care of patients with Soil transmitted helminths, including those with disseminated strongyloidiasis^37^ in whom oral administration of the ivermectin is not always successful^38,39^.

Another potential application is on the treatment or prevention of helminths currently considered not susceptible to ivermectin like *Schistosoma mansoni*, which expresses a variant of ivermectin’s primary target, the glutamate-gated chloride channels, that is only susceptible to a concentration of 1 μM, which is not readily attainable with oral doses but can be reached in the lung with a nebulized formulation.

Additionally, ivermectin’s anti-inflammatory properties may serve as primary or adjuvant therapy for respiratory diseases with an inflammatory component caused by chemical agents or pathogens^35,40^ as well as local and metastatic cancer^41^.

### Limitations of this study

This proof-of-concept study could be considered a pilot given the reduced number of animals used, other limitations include that a single measure from the lung concentration was taken at any given point which prevents the assessment of whether there is differential deposit within an individual lung or that there was no concurrent control for the toxicology of rats euthanized at 72 or 140 h. Additionally, the formulation was administered by nose and mouth exposure, hence, the lung levels achieved may not reflect those potentially achieved in a human with active inhalation.

We acknowledge that an ethanol-based formulation administered with a system that requires a high flow of oxygen was appropriate for proving the concept but not for scale up, further investment into formulation development would be needed to pursue this route of administration for any potential indication.

### Next steps

The finding that ivermectin concentrations with potential for pharmacodynamic effect were measured in the lungs seven days after inhaled therapy warrant additional research on the potential use of nebulized ivermectin for susceptible respiratory diseases including COVID-19. The immediate next step is the conduction of an extended animal model in rodent and non-rodent species to collect the additional safety data required for a first administration in humans and potentially, the use of animal models of infection to assess the efficacy of this therapy against relevant pathogens including SARS-CoV-2, helminths and possibly inflammatory as well as neoplastic diseases proper of the lung tissue.

## Conclusion

This study provides evidence that ivermectin can be delivered in high concentrations to rat lungs by nebulization, but this warrants additional research to determine safety and efficacy, not direct extrapolation to humans via compassionate use or accelerated approval.

## Supplementary information

Supplementary Information.

## Data Availability

All study data is contained within this manuscript and the supplementary material.
