# Supplementary material for: Nebulized ivermectin for COVID-19 and other respiratory diseases, a proof of concept, dose-ranging study in rats
Source: Sci Rep. 2020 Oct 13;10:17073. doi: 10.1038/s41598-020-74084-y (PMC7555481; doi:10.1038/s41598-020-74084-y)
Supplement: Supplementary file 1 — Supplementary Information. [file 41598_2020_74084_MOESM1_ESM.pdf]

Nebulized ivermectin for COVID-19 and other respiratory diseases, a proof of concept, dose-ranging study in rats

Carlos Chaccour, Gloria Abizanda, Angel Irigoyen-Barrio, Aina Casellas, Azucena Aldaz, Fernando Martínez-Galán, Felix Hammann, Ana Gloria Gil

Contents

Figure S1. Macroscopic aspect of the lungs of TM6, one male in the higher dose group. ... 2
Figure S2. Histologic image of the lungs of TM6 ... 3
Figure S3. Micromolar concentrations achieved in lung tissue stratified by dose administered and sex ... 4
Table S1. Serum biochemistry profile of all male rats ... 5
Table S2. Serum biochemistry profile of all female rats ... 6
Table S3. Modified Irwin Test: Parameter Grading System ... 7
Table S4. General symptomatology - High dose treatment group. Animal TM4 ... 8
Table S5. General symptomatology - High dose treatment group. Animal TM5 ... 9
Table S6. General symptomatology - High dose treatment group. Animal TM6 ... 10
Table S7. General symptomatology - High dose treatment group. Animal TF1 ... 11
Table S8. General symptomatology - High dose treatment group. Animal TF2 ... 12
Table S9. General symptomatology - High dose treatment group. Animal TF3 ... 13
Table S10. General symptomatology - low-dose treated group. Animal TM1 ... 14
Table S11. General symptomatology - low-dose treated group. Animal TM2 ... 15
Table S12. General symptomatology - low-dose treated group. Animal TM3 ... 16
Table S13. General symptomatology - low-dose treatment group. Animal TF4 ... 17
Table S14. General symptomatology - low-dose treated group. Animal TF5 ... 18
Table S15. General symptomatology - low-dose treatment group. Animal TF6 ... 19
Table S16. General symptomatology - Vehicle control group. Animal CVM1 ... 20
Table S17: General symptomatology - Vehicle control group. Animal CVF1 ... 21
Table S18. Adjusted ethanol dose and weight changes in male rats ... 22
Table S19. Adjusted ethanol dose and weight changes in female rats ... 23
Table S20. Full blood count of male rats ... 24
Table S21. Full blood count of female rats ... 25
Table S22. Absolute and relative weights of lungs and livers of male rats ... 26
Table S23. Absolute and relative weights of lungs and livers of female rats ... 27

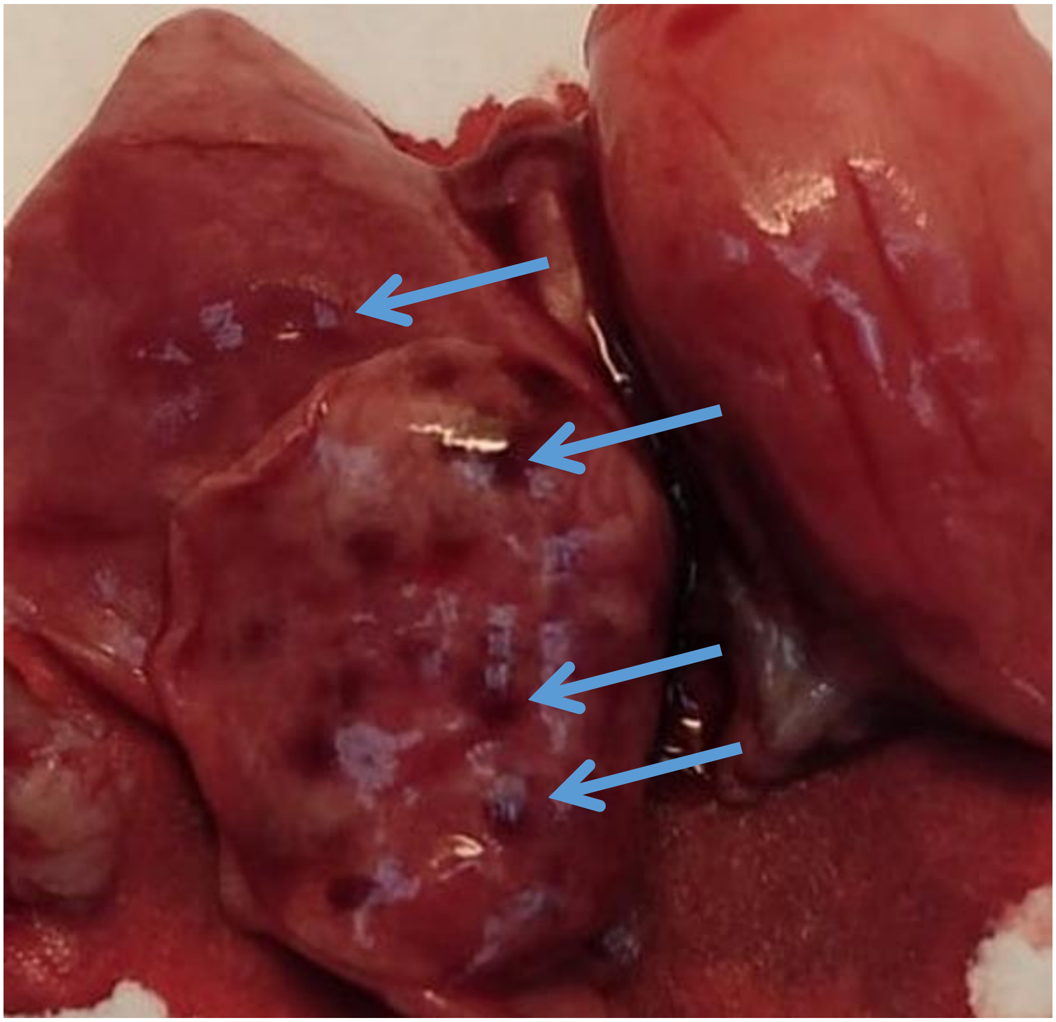

Figure S1. Macroscopic aspect of the lungs of TM6, one male in the higher dose group.

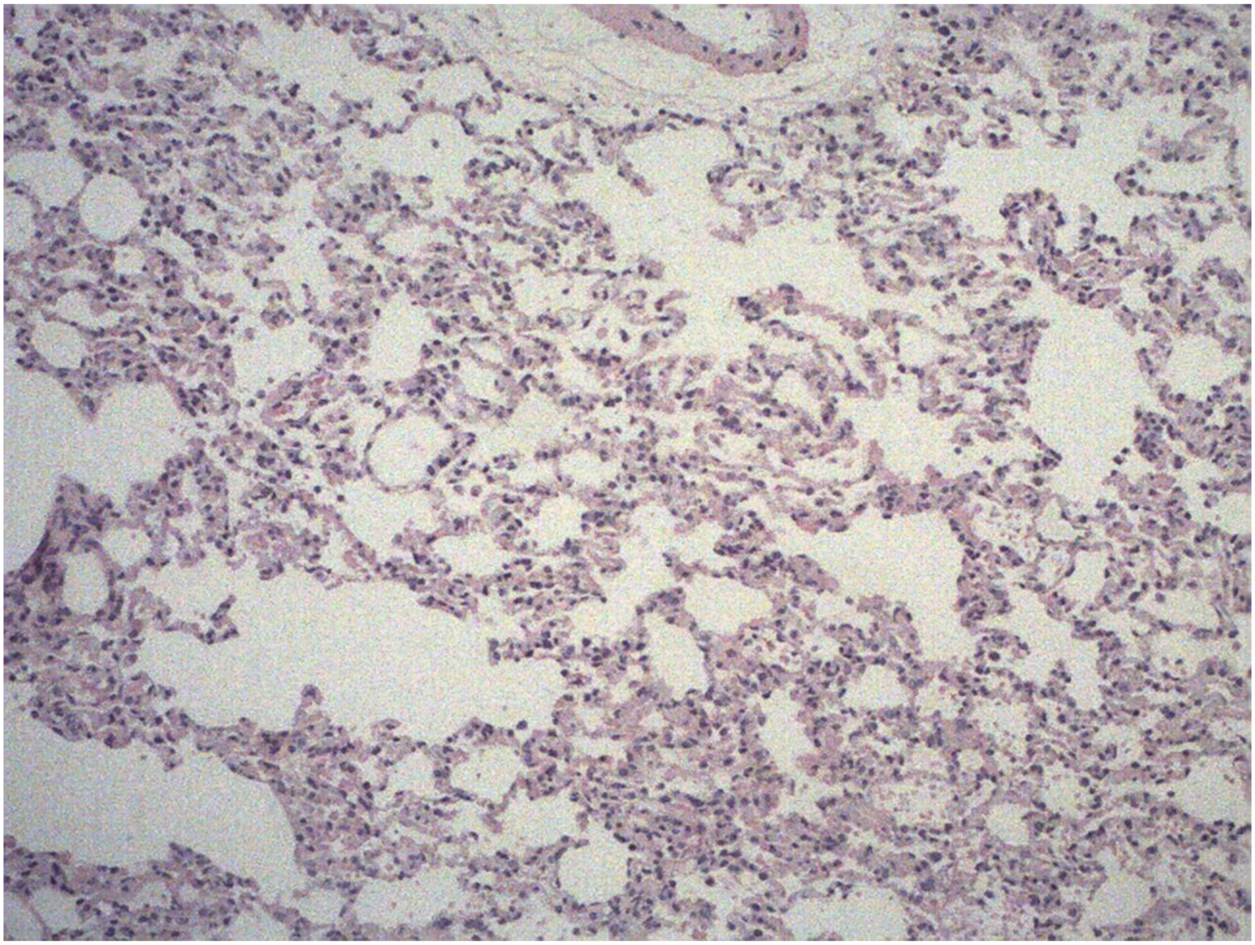

Figure S2. Histologic image of the lungs of TM6.

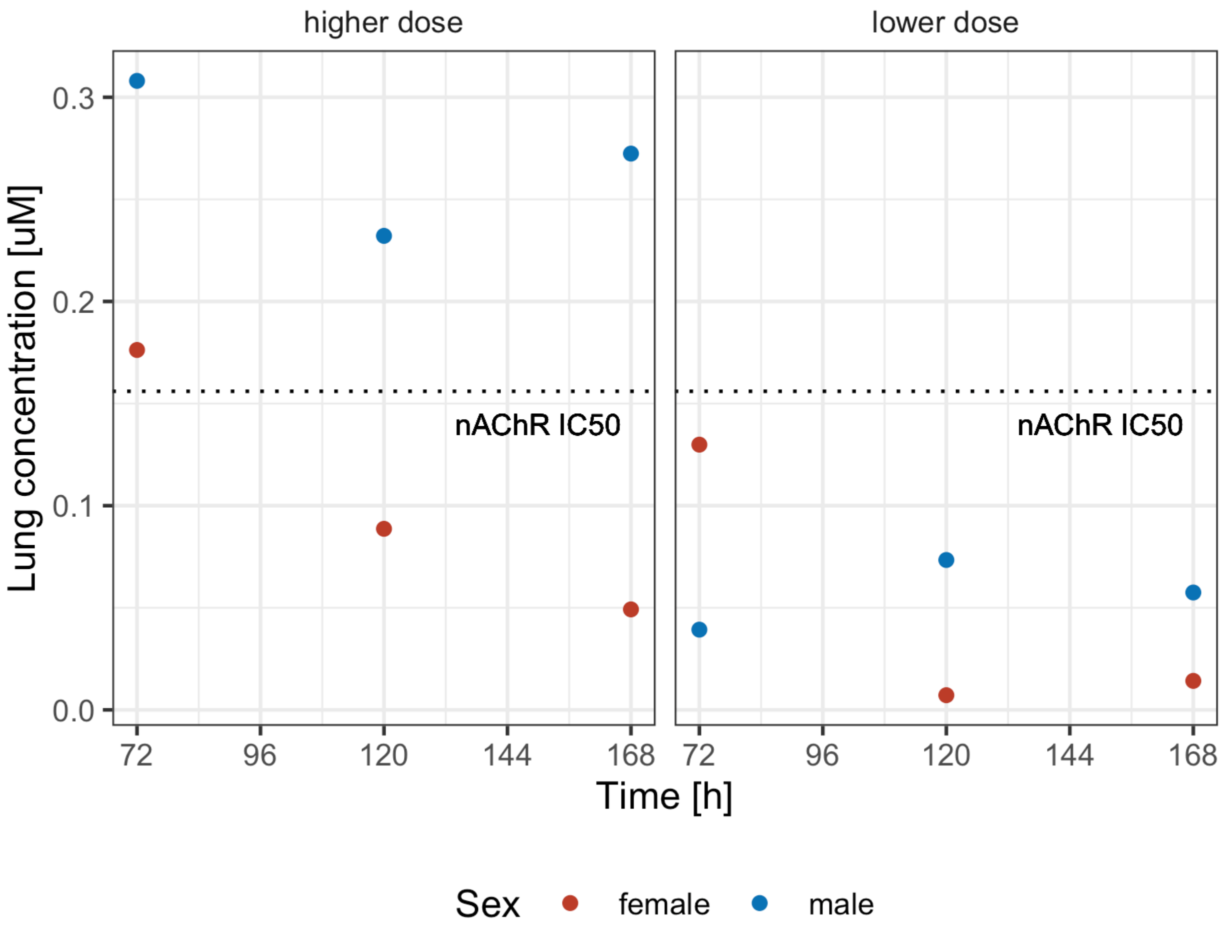

Figure S3. Micromolar concentrations achieved in lung tissue stratified by dose administered and sex (lower dose = 84-98 mg/kg, higher dose 106-140 mg/kg). nAChR IC50 value taken from [Krause 1997]

Subject	Weight	ivermectin dose (mg/kg)	Ethanol dose (g/kg)	time of biochemistry	Tot. Prot. (g/dL)	Alb (g/dL)	AST (U/L)	ALT (U/L)	Tot. Bil.* (mg/dL)	Tot. Cho. (mg/dL)	Glu. (mg/dL)	BUN (mg/dL)	Creat. (mg/dL)	CPK* (U/L)	LDH* (U/L)
					5.3-6.4	3.4-4.1	78-164	34-85	0.01-0.12	36-79	87-209	13-22	0.2-0.3	4-327	1-513
TM1	303.9	98.72	7.79	72 hours	5.5	3.5	**61**	35	0.06	77	**311**	17.7	**0.38**	205	249
TM2	333.2	90.04	7.11	168 hours	6.1	3.7	140	45	0.09	**97**	169	15.4	**0.35**	**1497**	**1816**
TM3	346.9	86.48	6.83	140 hours	5.8	3.8	**71**	41	0.04	**90**	198	20.1	**0.35**	297	398
TM4	386.9	108.56	6.12	72 hours	5.5	3.6	**71**	35	0.05	**81**	**235**	13.1	0.28	149	113
TM5	360.6	116.47	6.57	168 hours	6	3.9	106	48	0.06	**107**	117	14.9	**0.35**	**723**	**1270**
TM6	372.5	112.75	6.36	140 hours	6.1	4.1	**75**	54	0.04	**85**	**2040**	14.9	**0.37**	270	341
CVM1	331.5	0.00	7.14	168 hours	5.9	3.7	107	39	0.06	77	**223**	15.4	**0.37**	**760**	**1405**

All values from the animal provider https://www.envigo.com except those marked* taken from Lillie et al. Hum Exp Toxicol (1996) 15, 612-16.
Tot. Prot: total serum protein, Alb: serum albumin, AST: aspartate aminotransferase, ALT: alanin aminotransferase, Tot. Bil: total bilirrubin, Glu: gluchose, BUN: blood urea nitrogen, Creat: serum creatinin, CPK: creatin phosphokinase, LDH: lactate dehydrogenase

Table S1. Serum biochemistry profile of all male rats

Subject	Weight	ivermectin dose (mg/kg)	Ethanol dose (g/kg)	time of biochemistry	Tot. Prot. (g/dL)	Alb (g/dL)	AST (U/L)	ALT (U/L)	Tot. Bil.* (mg/dL)	Tot. Cho. (mg/dL)	Glu. (mg/dL)	BUN (mg/dL)	Creat. (mg/dL)	CPK* (U/L)	LDH* (U/L)
					5.6-7	3.7-4.8	70-158	28-60	0.02-0.12	48-101	81-181	12-23	0.2-0.4	1-327	1-600
TF1	211.6	141.78	11.19	72 hours	5.7	4	74	41	0.04	**189**	176	14.5	0.31	342	459
TF2	226.4	132.51	10.46	168 hours	5.8	4.2	**271**	**100**	0.06	89	**448**	18.7	**0.58**	**966**	**1881**
TF3	237	126.58	9.99	140 hours	5.8	4.1	97	47	0.06	90	**195**	15.4	0.39	**572**	**747**
TF4	218.8	95.98	10.82	72 hours	5.9	4.1	62	37	0.04	92	175	17.7	0.38	186	226
TF5	251.2	83.60	9.43	168 hours	5.2	**3.5**	143	42	0.02	86	**208**	14.0	0.33	**925**	**881**
TF6	238.4	88.09	9.93	140 hours	5.9	4.2	93	50	0.06	76	148	14.9	0.35	**494**	**775**
CVF1	249.5	0.00	9.49	168 hours	6.1	4.4	128	45	0.05	95	118	15.9	**0.41**	**1194**	**1744**

All values from the animal provider https://www.envigo.com except those marked* taken from Lillie et al. Hum Exp Toxicol (1996) 15, 612-16.
Tot. Prot: total serum protein, Alb: serum albumin, AST: aspartate aminotransferase, ALT: alanin aminotransferase, Tot. Bil: total bilirrubin, Glu: gluchose, BUN: blood urea nitrogen, Creat: serum creatinin, CPK: creatin phosphokinase, LDH: lactate dehydrogenase

Table S2. Serum biochemistry profile of all female rats

Studied parameters/ Normal grading		Rating
Visual location	4	The animal extends its front legs about 10-12 mm away from the contact patch.
Spontaneous activity	4	Vigorous, moderately fast movements
Reactivity	4	Animal on alert: agile and active head and body movements, but not exaggerated or abrupt. The animal walks around the cage "sniffing and sniffing", in an exploratory attitude
Response to contact	4	Rapid withdrawal on contact
Response to pain	4	Active withdrawal of the animal, fast, but not abruptly after pinching the tail
Response to scare	4	Sudden shaking of the animal at the snap of a finger
Stereotypes	0	Absence
Vocation	0	No screaming or shouting
Passivity	0	The animal opposes a pinch of skin between the ears, removes itself or escapes
Irritability	0	No attempts to scratch or bite the observer
Fear	0	The animal scans the finger, sniffs it, sniffs it repeatedly
Corridor	>10	The animal travels more than 10 cm of corridor
Body position	4	Animal on all four legs
Prey force	4	Moderate resistance
Body tone	4	Moderate resistance
Member Tone	4	Moderate resistance
Atrial Reflex	4	Retraction of the ear when approaching a tweezer
Corneal reflex	4	The animal closes its eyes or winks as it brings a pincer to the eye
Ipsilateral reflex	4	Removal of the animal actively, quickly, but not abruptly after clamping a phalanx
Balance	4	The animal places its hind legs quickly on the balance beam
Straightening reflex	0	The animal falls on all four legs
Tremors	0	Absence
Contractions	0	Absence
Ataxic walking	0	Absence
Hypotonic walking	0	Absence
Breast lift	0	Hind legs slightly bent so that the abdomen does not touch the ground
Straub's tail	0	Absence
Contortions	0	Absence
Pupil size	4	The pupil occupies ½ of the eyeball
Palpebral opening	4	Approximately ½ of closed eye
Skin color	4	Pink skin (not white)
Heart rate	4	Absence of bradycardia or tachycardia
Breathing rate	4	Between 60 and 200 times/minute
Exophthalmos	0	Absence
Micturition	0	Normal volume
Salivation	4	¼ of the wet submaxillary area
Piloerection	0	Absence
Diarrhoea	0	Absence
Hypothermia	0	No decrease in temperature detected

Table S3. Modified Irwin Test: Parameter Grading System

Studied parameters / Assigned evaluation		Period of observation			
		Before the administration	24 hours	48 hours	Day 3
Visual location	4	4	4	4	4
Spontaneous activity	4	4	4	4	4
Reactivity	4	4	4	4	4
Response to contact	4	4	4	4	4
Response to pain	4	4	4	4	4
Response to scare	4	4	4	4	4
Stereotypes	0	0	0	0	0
Vocation	0	0	0	0	0
Passivity	0	0	0	0	0
Irritability	0	0	0	0	0
Fear	0	0	0	0	0
Corridor	>10	>10	>10	>10	>10
Body position	4	4	4	4	4
Prey force	4	4	4	4	4
Body tone	4	4	4	4	4
Member Tone	4	4	4	4	4
Atrial Reflex	4	4	4	4	4
Corneal reflex	4	4	4	4	4
Ipsilateral reflex	4	4	4	4	4
Balance	4	4	4	4	4
Straightening reflex	0	0	0	0	0
Tremors	0	0	0	0	0
Contractions	0	0	0	0	0
Ataxic walking	0	0	0	0	0
Hypotonic walking	0	0	0	0	0
Breast lift	0	0	0	0	0
Straub's tail	0	0	0	0	0
Contortions	0	0	0	0	0
Pupil size	4	4	4	4	4
Palpebral opening	4	4	4	4	4
Skin color	4	4	4	4	4
Heart rate	4	4	4	4	4
Breathing rate	4	4	4	4	4
Exophthalmos	0	0	0	0	0
Micturition	0	0	0	0	0
Salivation	4	4	4	4	4
Piloerection	0	0	0	0	0
Diarrhoea	0	0	0	0	0
Hypothermia	0	0	0	0	0

Table S4. General symptomatology - High dose treatment group. Animal TM4
The table shows, for each established evaluation time (before the first administration and 24 hours, 48 hours; and 3 days after administration) the evaluation assigned to the TM4 animal (108 mg/kg). The shaded box contains the corresponding values with normal symptomatology for Sprague Dawley rats.

Studied parameters / Assigned evaluation		Period of observation							
		Before the administration	24 hours	48 hours	Day 3	Day 4	Day 5	Day 6	Day 7
Visual location	4	4	4	4	4	4	4	4	4
Spontaneous activity	4	4	4	4	4	4	4	4	4
Reactivity	4	4	4	4	4	4	4	4	4
Response to contact	4	4	4	4	4	4	4	4	4
Response to pain	4	4	4	4	4	4	4	4	4
Response to scare	4	4	4	4	4	4	4	4	4
Stereotypes	0	0	0	0	0	0	0	0	0
Vocation	0	0	0	0	0	0	0	0	0
Passivity	0	0	0	0	0	0	0	0	0
Irritability	0	0	0	0	0	0	0	0	0
Fear	0	0	0	0	0	0	0	0	0
Corridor	>10	>10	>10	>10	>10	>10	>10	>10	>10
Body position	4	4	4	4	4	4	4	4	4
Prey force	4	4	4	4	4	4	4	4	4
Body tone	4	4	4	4	4	4	4	4	4
Member Tone	4	4	4	4	4	4	4	4	4
Atrial Reflex	4	4	4	4	4	4	4	4	4
Corneal reflex	4	4	4	4	4	4	4	4	4
Ipsilateral reflex	4	4	4	4	4	4	4	4	4
Balance	4	4	4	4	4	4	4	4	4
Straightening reflex	0	0	0	0	0	0	0	0	0
Tremors	0	0	0	0	0	0	0	0	0
Contractions	0	0	0	0	0	0	0	0	0
Ataxic walking	0	0	0	0	0	0	0	0	0
Hypotonic walking	0	0	0	0	0	0	0	0	0
Breast lift	0	0	0	0	0	0	0	0	0
Straub's tail	0	0	0	0	0	0	0	0	0
Contortions	0	0	0	0	0	0	0	0	0
Pupil size	4	4	4	4	4	4	4	4	4
Palpebral opening	4	4	4	4	4	4	4	4	4
Skin color	4	4	4	4	4	4	4	4	4
Heart rate	4	4	4	4	4	4	4	4	4
Breathing rate	4	4	4	4	4	4	4	4	4
Exophthalmos	0	0	0	0	0	0	0	0	0
Micturition	0	0	0	0	0	0	0	0	0
Salivation	4	4	4	4	4	4	4	4	4
Piloerection	0	0	0	0	0	0	0	0	0
Diarrhoea	0	0	0	0	0	0	0	0	0
Hypothermia	0	0	0	0	0	0	0	0	0

Table S5. General symptomatology - High dose treatment group. Animal TM5
The table shows, for each established evaluation time (before the first administration and 24 hours, 48 hours; and 3, 4, 5, 6 and 7 days after administration) the evaluation assigned to the TM5 animal (116 mg/kg). The shaded box contains the corresponding values with normal symptomatology for Sprague Dawley rats.

Studied parameters / Assigned evaluation		Period of observation					
		Before the administration	24 hours	48 hours	Day 3	Day 4	Day 5
Visual location	4	4	4	4	4	4	4
Spontaneous activity	4	4	4	4	4	4	4
Reactivity	4	4	4	4	4	4	4
Response to contact	4	4	4	4	4	4	4
Response to pain	4	4	4	4	4	4	4
Response to scare	4	4	4	4	4	4	4
Stereotypes	0	0	0	0	0	0	0
Vocation	0	0	0	0	0	0	0
Passivity	0	0	0	0	0	0	0
Irritability	0	0	0	0	0	0	0
Fear	0	0	0	0	0	0	0
Corridor	>10	>10	>10	>10	>10	>10	>10
Body position	4	4	4	4	4	4	4
Prey force	4	4	4	4	4	4	4
Body tone	4	4	4	4	4	4	4
Member Tone	4	4	4	4	4	4	4
Atrial Reflex	4	4	4	4	4	4	4
Corneal reflex	4	4	4	4	4	4	4
Ipsilateral reflex	4	4	4	4	4	4	4
Balance	4	4	4	4	4	4	4
Straightening reflex	0	0	0	0	0	0	0
Tremors	0	0	0	0	0	0	0
Contractions	0	0	0	0	0	0	0
Ataxic walking	0	0	0	0	0	0	0
Hypotonic walking	0	0	0	0	0	0	0
Breast lift	0	0	0	0	0	0	0
Straub's tail	0	0	0	0	0	0	0
Contortions	0	0	0	0	0	0	0
Pupil size	4	4	4	4	4	4	4
Palpebral opening	4	4	4	4	4	4	4
Skin color	4	4	4	4	4	4	4
Heart rate	4	4	4	4	4	4	4
Breathing rate	4	4	4	4	4	4	4
Exophthalmos	0	0	0	0	0	0	0
Micturition	0	0	0	0	0	0	0
Salivation	4	4	4	4	4	4	4
Piloerection	0	0	0	0	0	0	0
Diarrhoea	0	0	0	0	0	0	0
Hypothermia	0	0	0	0	0	0	0

Table S6. General symptomatology - High dose treatment group. Animal TM6
The table shows, for each established evaluation time (before the first administration and 24 hours, 48 hours; and 3, 4 and 5 days after administration) the evaluation assigned to the animal TM6 (112 mg/kg). The shaded box contains the corresponding values with normal symptomatology for Sprague Dawley rats.

Studied parameters / Assigned evaluation		Period of observation			
		Before the administration	24 hours	48 hours	Day 3
Visual location	4	4	4	4	4
Spontaneous activity	4	4	4	4	4
Reactivity	4	4	4	4	4
Response to contact	4	4	4	4	4
Response to pain	4	4	4	4	4
Response to scare	4	4	4	4	4
Stereotypes	0	0	0	0	0
Vocation	0	0	0	0	0
Passivity	0	0	0	0	0
Irritability	0	0	0	0	0
Fear	0	0	0	0	0
Corridor	>10	>10	>10	>10	>10
Body position	4	4	4	4	4
Prey force	4	4	4	4	4
Body tone	4	4	4	4	4
Member Tone	4	4	4	4	4
Atrial Reflex	4	4	4	4	4
Corneal reflex	4	4	4	4	4
Ipsilateral reflex	4	4	4	4	4
Balance	4	4	4	4	4
Straightening reflex	0	0	0	0	0
Tremors	0	0	0	0	0
Contractions	0	0	0	0	0
Ataxic walking	0	0	0	0	0
Hypotonic walking	0	0	0	0	0
Breast lift	0	0	0	0	0
Straub's tail	0	0	0	0	0
Contortions	0	0	0	0	0
Pupil size	4	4	4	4	4
Palpebral opening	4	4	4	4	4
Skin color	4	4	4	4	4
Heart rate	4	4	4	4	4
Breathing rate	4	4	4	4	4
Exophthalmos	0	0	0	0	0
Micturition	0	0	0	0	0
Salivation	4	4	4	4	4
Pilot	0	0	0	0	0
Diarrhoea	0	0	0	0	0
Hypothermia	0	0	0	0	0

Table S7. General symptomatology - High dose treatment group. Animal TF1
The table shows, for each established evaluation time (before the first administration and 24 hours, 48 hours; and 3 days after administration) the evaluation assigned to the TF1 animal (141 mg/kg). The shaded box contains the corresponding values with normal symptomatology for Sprague Dawley rats.

Studied parameters / Assigned evaluation		Period of observation							
		Before the administration	24 hours	48 hours	Day 3	Day 4	Day 5	Day 6	Day 7
Visual location	4	4	4	4	4	4	4	4	4
Spontaneous activity	4	4	4	4	4	4	4	4	4
Reactivity	4	4	4	4	4	4	4	4	4
Response to contact	4	4	4	4	4	4	4	4	4
Response to pain	4	4	4	4	4	4	4	4	4
Response to scare	4	4	4	4	4	4	4	4	4
Stereotypes	0	0	0	0	0	0	0	0	0
Vocation	0	0	0	0	0	0	0	0	0
Passivity	0	0	0	0	0	0	0	0	0
Irritability	0	0	0	0	0	0	0	0	0
Fear	0	0	0	0	0	0	0	0	0
Corridor	>10	>10	>10	>10	>10	>10	>10	>10	>10
Body position	4	4	4	4	4	4	4	4	4
Prey force	4	4	4	4	4	4	4	4	4
Body tone	4	4	4	4	4	4	4	4	4
Member Tone	4	4	4	4	4	4	4	4	4
Atrial Reflex	4	4	4	4	4	4	4	4	4
Corneal reflex	4	4	4	4	4	4	4	4	4
Ipsilateral reflex	4	4	4	4	4	4	4	4	4
Balance	4	4	4	4	4	4	4	4	4
Straightening reflex	0	0	0	0	0	0	0	0	0
Tremors	0	0	0	0	0	0	0	0	0
Contractions	0	0	0	0	0	0	0	0	0
Ataxic walking	0	0	0	0	0	0	0	0	0
Hypotonic walking	0	0	0	0	0	0	0	0	0
Breast lift	0	0	0	0	0	0	0	0	0
Straub's tail	0	0	0	0	0	0	0	0	0
Contortions	0	0	0	0	0	0	0	0	0
Pupil size	4	4	4	4	4	4	4	4	4
Palpebral opening	4	4	4	4	4	4	4	4	4
Skin color	4	4	4	4	4	4	4	4	4
Heart rate	4	4	4	4	4	4	4	4	4
Breathing rate	4	4	4	4	4	4	4	4	4
Exophthalmos	0	0	0	0	0	0	0	0	0
Micturition	0	0	0	0	0	0	0	0	0
Salivation	4	4	4	4	4	4	4	4	4
Piloerection	0	0	0	0	0	0	0	0	0
Diarrhoea	0	0	0	0	0	0	0	0	0
Hypothermia	0	0	0	0	0	0	0	0	0

Table S8. General symptomatology - High dose treatment group. Animal TF2
The table shows, for each established evaluation time (before the first administration and 24 hours, 48 hours; and 3, 4, 5, 6 and 7 days after administration) the evaluation assigned to the TF3 animal (132 mg/kg). The shaded box contains the corresponding values with normal symptomatology for Sprague Dawley rats.

Studied parameters / Assigned evaluation		Period of observation					
		Before the administration	24 hours	48 hours	Day 3	Day 4	Day 5
Visual location	4	4	4	4	4	4	4
Spontaneous activity	4	4	4	4	4	4	4
Reactivity	4	4	4	4	4	4	4
Response to contact	4	4	4	4	4	4	4
Response to pain	4	4	4	4	4	4	4
Response to scare	4	4	4	4	4	4	4
Stereotypes	0	0	0	0	0	0	0
Vocation	0	0	0	0	0	0	0
Passivity	0	0	0	0	0	0	0
Irritability	0	0	0	0	0	0	0
Fear	0	0	0	0	0	0	0
Corridor	>10	>10	>10	>10	>10	>10	>10
Body position	4	4	4	4	4	4	4
Prey force	4	4	4	4	4	4	4
Body tone	4	4	4	4	4	4	4
Member Tone	4	4	4	4	4	4	4
Atrial Reflex	4	4	4	4	4	4	4
Corneal reflex	4	4	4	4	4	4	4
Ipsilateral reflex	4	4	4	4	4	4	4
Balance	4	4	4	4	4	4	4
Straightening reflex	0	0	0	0	0	0	0
Tremors	0	0	0	0	0	0	0
Contractions	0	0	0	0	0	0	0
Ataxic walking	0	0	0	0	0	0	0
Hypotonic walking	0	0	0	0	0	0	0
Breast lift	0	0	0	0	0	0	0
Straub's tail	0	0	0	0	0	0	0
Contortions	0	0	0	0	0	0	0
Pupil size	4	4	4	4	4	4	4
Palpebral opening	4	4	4	4	4	4	4
Skin color	4	4	4	4	4	4	4
Heart rate	4	4	4	4	4	4	4
Breathing rate	4	4	4	4	4	4	4
Exophthalmos	0	0	0	0	0	0	0
Micturition	0	0	0	0	0	0	0
Salivation	4	4	4	4	4	4	4
Piloerection	0	0	0	0	0	0	0
Diarrhoea	0	0	0	0	0	0	0
Hypothermia	0	0	0	0	0	0	0

Table S9. General symptomatology - High dose treatment group. Animal TF3
The table shows, for each established evaluation time (before the first administration and 24 hours, 48 hours; and 3, 4 and 5 days after administration) the evaluation assigned to the TF3 animal (126 mg/kg). The shaded box contains the corresponding values with normal symptomatology for Sprague Dawley rats.

Studied parameters / Assigned evaluation		Period of observation			
		Before the administration	24 hours	48 hours	Day 3
Visual location	4	4	4	4	4
Spontaneous activity	4	4	4	4	4
Reactivity	4	4	4	4	4
Response to contact	4	4	4	4	4
Response to pain	4	4	4	4	4
Response to scare	4	4	4	4	4
Stereotypes	0	0	0	0	0
Vocation	0	0	0	0	0
Passivity	0	0	0	0	0
Irritability	0	0	0	0	0
Fear	0	0	0	0	0
Corridor	>10	>10	>10	>10	>10
Body position	4	4	4	4	4
Prey force	4	4	4	4	4
Body tone	4	4	4	4	4
Member Tone	4	4	4	4	4
Atrial Reflex	4	4	4	4	4
Corneal reflex	4	4	4	4	4
Ipsilateral reflex	4	4	4	4	4
Balance	4	4	4	4	4
Straightening reflex	0	0	0	0	0
Tremors	0	0	0	0	0
Contractions	0	0	0	0	0
Ataxic walking	0	0	0	0	0
Hypotonic walking	0	0	0	0	0
Breast lift	0	0	0	0	0
Straub's tail	0	0	0	0	0
Contortions	0	0	0	0	0
Pupil size	4	4	4	4	4
Palpebral opening	4	4	4	4	4
Skin color	4	4	4	4	4
Heart rate	4	4	4	4	4
Breathing rate	4	4	4	4	4
Exophthalmos	0	0	0	0	0
Micturition	0	0	0	0	0
Salivation	4	4	4	4	4
Piloerection	0	0	0	0	0
Diarrhoea	0	0	0	0	0
Hypothermia	0	0	0	0	0

Table S10. General symptomatology - low-dose treated group. Animal TM1
The table shows, for each established evaluation time (before the first administration and 24 hours, 48 hours; and 3 days after administration) the evaluation assigned to the animal TM1 (98 mg/kg). The shaded box contains the corresponding values with normal symptomatology for Sprague Dawley rats.

Studied parameters / Assigned evaluation		Period of observation							
		Before the administration	24 hours	48 hours	Day 3	Day 4	Day 5	Day 6	Day 7
Visual location	4	4	4	4	4	4	4	4	4
Spontaneous activity	4	4	4	4	4	4	4	4	4
Reactivity	4	4	4	4	4	4	4	4	4
Response to contact	4	4	4	4	4	4	4	4	4
Response to pain	4	4	4	4	4	4	4	4	4
Response to scare	4	4	4	4	4	4	4	4	4
Stereotypes	0	0	0	0	0	0	0	0	0
Vocation	0	0	0	0	0	0	0	0	0
Passivity	0	0	0	0	0	0	0	0	0
Irritability	0	0	0	0	0	0	0	0	0
Fear	0	0	0	0	0	0	0	0	0
Corridor	>10	>10	>10	>10	>10	>10	>10	>10	>10
Body position	4	4	4	4	4	4	4	4	4
Prey force	4	4	4	4	4	4	4	4	4
Body tone	4	4	4	4	4	4	4	4	4
Member Tone	4	4	4	4	4	4	4	4	4
Atrial Reflex	4	4	4	4	4	4	4	4	4
Corneal reflex	4	4	4	4	4	4	4	4	4
Ipsilateral reflex	4	4	4	4	4	4	4	4	4
Balance	4	4	4	4	4	4	4	4	4
Straightening reflex	0	0	0	0	0	0	0	0	0
Tremors	0	0	0	0	0	0	0	0	0
Contractions	0	0	0	0	0	0	0	0	0
Ataxic walking	0	0	0	0	0	0	0	0	0
Hypotonic walking	0	0	0	0	0	0	0	0	0
Breast lift	0	0	0	0	0	0	0	0	0
Straub's tail	0	0	0	0	0	0	0	0	0
Contortions	0	0	0	0	0	0	0	0	0
Pupil size	4	4	4	4	4	4	4	4	4
Palpebral opening	4	4	4	4	4	4	4	4	4
Skin color	4	4	4	4	4	4	4	4	4
Heart rate	4	4	4	4	4	4	4	4	4
Breathing rate	4	4	4	4	4	4	4	4	4
Exophthalmos	0	0	0	0	0	0	0	0	0
Micturition	0	0	0	0	0	0	0	0	0
Salivation	4	4	4	4	4	4	4	4	4
Piloerection	0	0	0	0	0	0	0	0	0
Diarrhoea	0	0	0	0	0	0	0	0	0
Hypothermia	0	0	0	0	0	0	0	0	0

Table S11. General symptomatology - low-dose treated group. Animal TM2
The table shows, for each established evaluation time (before the first administration and 24 hours, 48 hours; and 3, 4, 5, 6 and 7 days after administration) the evaluation assigned to the TM2 animal (90 mg/kg). The shaded box contains the corresponding values with normal symptomatology for Sprague Dawley rats.

Studied parameters / Assigned evaluation		Period of observation					
		Before the administration	24 hours	48 hours	Day 3	Day 4	Day 5
Visual location	4	4	4	4	4	4	4
Spontaneous activity	4	4	4	4	4	4	4
Reactivity	4	4	4	4	4	4	4
Response to contact	4	4	4	4	4	4	4
Response to pain	4	4	4	4	4	4	4
Response to scare	4	4	4	4	4	4	4
Stereotypes	0	0	0	0	0	0	0
Vocation	0	0	0	0	0	0	0
Passivity	0	0	0	0	0	0	0
Irritability	0	0	0	0	0	0	0
Fear	0	0	0	0	0	0	0
Corridor	>10	>10	>10	>10	>10	>10	>10
Body position	4	4	4	4	4	4	4
Prey force	4	4	4	4	4	4	4
Body tone	4	4	4	4	4	4	4
Member Tone	4	4	4	4	4	4	4
Atrial Reflex	4	4	4	4	4	4	4
Corneal reflex	4	4	4	4	4	4	4
Ipsilateral reflex	4	4	4	4	4	4	4
Balance	4	4	4	4	4	4	4
Straightening reflex	0	0	0	0	0	0	0
Tremors	0	0	0	0	0	0	0
Contractions	0	0	0	0	0	0	0
Ataxic walking	0	0	0	0	0	0	0
Hypotonic walking	0	0	0	0	0	0	0
Breast lift	0	0	0	0	0	0	0
Straub's tail	0	0	0	0	0	0	0
Contortions	0	0	0	0	0	0	0
Pupil size	4	4	4	4	4	4	4
Palpebral opening	4	4	4	4	4	4	4
Skin color	4	4	4	4	4	4	4
Heart rate	4	4	4	4	4	4	4
Breathing rate	4	4	4	4	4	4	4
Exophthalmos	0	0	0	0	0	0	0
Micturition	0	0	0	0	0	0	0
Salivation	4	4	4	4	4	4	4
Piloerection	0	0	0	0	0	0	0
Diarrhoea	0	0	0	0	0	0	0
Hypothermia	0	0	0	0	0	0	0

Table S12. General symptomatology - low-dose treated group. Animal TM3
The table shows, for each established evaluation time (before the first administration and 24 hours, 48 hours; and 3, 4 and 5 days after administration) the evaluation assigned to the animal TM3 (86 mg/kg). The shaded box contains the corresponding values with normal symptomatology for Sprague Dawley rats.

Studied parameters / Assigned evaluation		Period of observation			
		Before the administration	24 hours	48 hours	Day 3
Visual location	4	4	4	4	4
Spontaneous activity	4	4	4	4	4
Reactivity	4	4	4	4	4
Response to contact	4	4	4	4	4
Response to pain	4	4	4	4	4
Response to scare	4	4	4	4	4
Stereotypes	0	0	0	0	0
Vocation	0	0	0	0	0
Passivity	0	0	0	0	0
Irritability	0	0	0	0	0
Fear	0	0	0	0	0
Corridor	>10	>10	>10	>10	>10
Body position	4	4	4	4	4
Prey force	4	4	4	4	4
Body tone	4	4	4	4	4
Member Tone	4	4	4	4	4
Atrial Reflex	4	4	4	4	4
Corneal reflex	4	4	4	4	4
Ipsilateral reflex	4	4	4	4	4
Balance	4	4	4	4	4
Straightening reflex	0	0	0	0	0
Tremors	0	0	0	0	0
Contractions	0	0	0	0	0
Ataxic walking	0	0	0	0	0
Hypotonic walking	0	0	0	0	0
Breast lift	0	0	0	0	0
Straub's tail	0	0	0	0	0
Contortions	0	0	0	0	0
Pupil size	4	4	4	4	4
Palpebral opening	4	4	4	4	4
Skin color	4	4	4	4	4
Heart rate	4	4	4	4	4
Breathing rate	4	4	4	4	4
Exophthalmos	0	0	0	0	0
Micturition	0	0	0	0	0
Salivation	4	4	4	4	4
Piloerection	0	0	0	0	0
Diarrhoea	0	0	0	0	0
Hypothermia	0	0	0	0	0

Table S13. General symptomatology - low-dose treatment group. Animal TF4
The table shows, for each established evaluation time (before the first administration and 24 hours, 48 hours; and 3 days after administration) the evaluation assigned to the TF4 animal (96 mg/kg). The shaded box contains the corresponding values with normal symptomatology for Sprague Dawley rats.

Studied parameters / Assigned evaluation		Period of observation							
		Before the administration	24 hours	48 hours	Day 3	Day 4	Day 5	Day 6	Day 7
Visual location	4	4	4	4	4	4	4	4	4
Spontaneous activity	4	4	4	4	4	4	4	4	4
Reactivity	4	4	4	4	4	4	4	4	4
Response to contact	4	4	4	4	4	4	4	4	4
Response to pain	4	4	4	4	4	4	4	4	4
Response to scare	4	4	4	4	4	4	4	4	4
Stereotypes	0	0	0	0	0	0	0	0	0
Vocation	0	0	0	0	0	0	0	0	0
Passivity	0	0	0	0	0	0	0	0	0
Irritability	0	0	0	0	0	0	0	0	0
Fear	0	0	0	0	0	0	0	0	0
Corridor	>10	>10	>10	>10	>10	>10	>10	>10	>10
Body position	4	4	4	4	4	4	4	4	4
Prey force	4	4	4	4	4	4	4	4	4
Body tone	4	4	4	4	4	4	4	4	4
Member Tone	4	4	4	4	4	4	4	4	4
Atrial Reflex	4	4	4	4	4	4	4	4	4
Corneal reflex	4	4	4	4	4	4	4	4	4
Ipsilateral reflex	4	4	4	4	4	4	4	4	4
Balance	4	4	4	4	4	4	4	4	4
Straightening reflex	0	0	0	0	0	0	0	0	0
Tremors	0	0	0	0	0	0	0	0	0
Contractions	0	0	0	0	0	0	0	0	0
Ataxic walking	0	0	0	0	0	0	0	0	0
Hypotonic walking	0	0	0	0	0	0	0	0	0
Breast lift	0	0	0	0	0	0	0	0	0
Straub's tail	0	0	0	0	0	0	0	0	0
Contortions	0	0	0	0	0	0	0	0	0
Pupil size	4	4	4	4	4	4	4	4	4
Palpebral opening	4	4	4	4	4	4	4	4	4
Skin color	4	4	4	4	4	4	4	4	4
Heart rate	4	4	4	4	4	4	4	4	4
Breathing rate	4	4	4	4	4	4	4	4	4
Exophthalmos	0	0	0	0	0	0	0	0	0
Micturition	0	0	0	0	0	0	0	0	0
Salivation	4	4	4	4	4	4	4	4	4
Piloerection	0	0	0	0	0	0	0	0	0
Diarrhoea	0	0	0	0	0	0	0	0	0
Hypothermia	0	0	0	0	0	0	0	0	0

Table S14. General symptomatology - low-dose treated group. Animal TF5
The table shows, for each established evaluation time (before the first administration and 24 hours, 48 hours; and 3, 4, 5, 6 and 7 days after administration) the evaluation assigned to the TF5 animal (83 mg/kg). The shaded box contains the corresponding values with normal symptomatology for Sprague Dawley rats.

Studied parameters / Assigned evaluation		Period of observation					
		Before the administration	24 hours	48 hours	Day 3	Day 4	Day 5
Visual location	4	4	4	4	4	4	4
Spontaneous activity	4	4	4	4	4	4	4
Reactivity	4	4	4	4	4	4	4
Response to contact	4	4	4	4	4	4	4
Response to pain	4	4	4	4	4	4	4
Response to scare	4	4	4	4	4	4	4
Stereotypes	0	0	0	0	0	0	0
Vocation	0	0	0	0	0	0	0
Passivity	0	0	0	0	0	0	0
Irritability	0	0	0	0	0	0	0
Fear	0	0	0	0	0	0	0
Corridor	>10	>10	>10	>10	>10	>10	>10
Body position	4	4	4	4	4	4	4
Prey force	4	4	4	4	4	4	4
Body tone	4	4	4	4	4	4	4
Member Tone	4	4	4	4	4	4	4
Atrial Reflex	4	4	4	4	4	4	4
Corneal reflex	4	4	4	4	4	4	4
Ipsilateral reflex	4	4	4	4	4	4	4
Balance	4	4	4	4	4	4	4
Straightening reflex	0	0	0	0	0	0	0
Tremors	0	0	0	0	0	0	0
Contractions	0	0	0	0	0	0	0
Ataxic walking	0	0	0	0	0	0	0
Hypotonic walking	0	0	0	0	0	0	0
Breast lift	0	0	0	0	0	0	0
Straub's tail	0	0	0	0	0	0	0
Contortions	0	0	0	0	0	0	0
Pupil size	4	4	4	4	4	4	4
Palpebral opening	4	4	4	4	4	4	4
Skin color	4	4	4	4	4	4	4
Heart rate	4	4	4	4	4	4	4
Breathing rate	4	4	4	4	4	4	4
Exophthalmos	0	0	0	0	0	0	0
Micturition	0	0	0	0	0	0	0
Salivation	4	4	4	4	4	4	4
Piloerection	0	0	0	0	0	0	0
Diarrhoea	0	0	0	0	0	0	0
Hypothermia	0	0	0	0	0	0	0

Table S15. General symptomatology - low-dose treatment group. Animal TF6
The table shows, for each established evaluation time (before the first administration and 24 hours, 48 hours; and 3, 4 and 5 days after administration) the evaluation assigned to the TF6 animal (88 mg/kg). The shaded box contains the corresponding values with normal symptomatology for Sprague Dawley rats.

Studied parameters / Assigned evaluation		Period of observation							
		Before the administration	24 hours	48 hours	Day 3	Day 4	Day 5	Day 6	Day 7
Visual location	4	4	4	4	4	4	4	4	4
Spontaneous activity	4	4	4	4	4	4	4	4	4
Reactivity	4	4	4	4	4	4	4	4	4
Response to contact	4	4	4	4	4	4	4	4	4
Response to pain	4	4	4	4	4	4	4	4	4
Response to scare	4	4	4	4	4	4	4	4	4
Stereotypes	0	0	0	0	0	0	0	0	0
Vocation	0	0	0	0	0	0	0	0	0
Passivity	0	0	0	0	0	0	0	0	0
Irritability	0	0	0	0	0	0	0	0	0
Fear	0	0	0	0	0	0	0	0	0
Corridor	>10	>10	>10	>10	>10	>10	>10	>10	>10
Body position	4	4	4	4	4	4	4	4	4
Prey force	4	4	4	4	4	4	4	4	4
Body tone	4	4	4	4	4	4	4	4	4
Member Tone	4	4	4	4	4	4	4	4	4
Atrial Reflex	4	4	4	4	4	4	4	4	4
Corneal reflex	4	4	4	4	4	4	4	4	4
Ipsilateral reflex	4	4	4	4	4	4	4	4	4
Balance	4	4	4	4	4	4	4	4	4
Straightening reflex	0	0	0	0	0	0	0	0	0
Tremors	0	0	0	0	0	0	0	0	0
Contractions	0	0	0	0	0	0	0	0	0
Ataxic walking	0	0	0	0	0	0	0	0	0
Hypotonic walking	0	0	0	0	0	0	0	0	0
Breast lift	0	0	0	0	0	0	0	0	0
Straub's tail	0	0	0	0	0	0	0	0	0
Contortions	0	0	0	0	0	0	0	0	0
Pupil size	4	4	4	4	4	4	4	4	4
Palpebral opening	4	4	4	4	4	4	4	4	4
Skin color	4	4	4	4	4	4	4	4	4
Heart rate	4	4	4	4	4	4	4	4	4
Breathing rate	4	4	4	4	4	4	4	4	4
Exophthalmos	0	0	0	0	0	0	0	0	0
Micturition	0	0	0	0	0	0	0	0	0
Salivation	4	4	4	4	4	4	4	4	4
Piloerection	0	0	0	0	0	0	0	0	0
Diarrhoea	0	0	0	0	0	0	0	0	0
Hypothermia	0	0	0	0	0	0	0	0	0

Table S16. General symptomatology - Vehicle control group. Animal CVM1
The table shows, for each established evaluation time (before the first administration and 24 hours, 48 hours; and 3, 4, 5, 6 and 7 days after administration) the evaluation assigned to the CVM1 animal. The shaded box contains the corresponding values with normal symptomatology for Sprague Dawley rats.

Studied parameters / Assigned evaluation		Period of observation							
		Before the administration	24 hours	48 hours	Day 3	Day 4	Day 5	Day 6	Day 7
Visual location	4	4	4	4	4	4	4	4	4
Spontaneous activity	4	4	4	4	4	4	4	4	4
Reactivity	4	4	4	4	4	4	4	4	4
Response to contact	4	4	4	4	4	4	4	4	4
Response to pain	4	4	4	4	4	4	4	4	4
Response to scare	4	4	4	4	4	4	4	4	4
Stereotypes	0	0	0	0	0	0	0	0	0
Vocation	0	0	0	0	0	0	0	0	0
Passivity	0	0	0	0	0	0	0	0	0
Irritability	0	0	0	0	0	0	0	0	0
Fear	0	0	0	0	0	0	0	0	0
Corridor	>10	>10	>10	>10	>10	>10	>10	>10	>10
Body position	4	4	4	4	4	4	4	4	4
Prey force	4	4	4	4	4	4	4	4	4
Body tone	4	4	4	4	4	4	4	4	4
Member Tone	4	4	4	4	4	4	4	4	4
Atrial Reflex	4	4	4	4	4	4	4	4	4
Corneal reflex	4	4	4	4	4	4	4	4	4
Ipsilateral reflex	4	4	4	4	4	4	4	4	4
Balance	4	4	4	4	4	4	4	4	4
Straightening reflex	0	0	0	0	0	0	0	0	0
Tremors	0	0	0	0	0	0	0	0	0
Contractions	0	0	0	0	0	0	0	0	0
Ataxic walking	0	0	0	0	0	0	0	0	0
Hypotonic walking	0	0	0	0	0	0	0	0	0
Breast lift	0	0	0	0	0	0	0	0	0
Straub's tail	0	0	0	0	0	0	0	0	0
Contortions	0	0	0	0	0	0	0	0	0
Pupil size	4	4	4	4	4	4	4	4	4
Palpebral opening	4	4	4	4	4	4	4	4	4
Skin color	4	4	4	4	4	4	4	4	4
Heart rate	4	4	4	4	4	4	4	4	4
Breathing rate	4	4	4	4	4	4	4	4	4
Exophthalmos	0	0	0	0	0	0	0	0	0
Micturition	0	0	0	0	0	0	0	0	0
Salivation	4	4	4	4	4	4	4	4	4
Piloerection	0	0	0	0	0	0	0	0	0
Diarrhoea	0	0	0	0	0	0	0	0	0
Hypothermia	0	0	0	0	0	0	0	0	0

Table S17: General symptomatology - Vehicle control group. Animal CVF1
The table shows, for each established evaluation time (before the first administration and 24 hours, 48 hours; and 3, 4, 5, 6 and 7 days after administration) the evaluation assigned to the FVC1 animal. The shaded box contains the corresponding values with normal symptomatology for Sprague Dawley rats.

Subject	ivermectin dose (mg/kg)	Ethanol dose (gr/kg)	Baseline Weight (g)	D3 Weight (g)	D5 Weight (g)	D7 Weight (g)
			**321-617**			
TM1	98.72	7.79	**303.9**	305.3	--	--
TM2	90.04	7.11	333.2	**323.9**	--	330.7
TM3	86.48	6.83	346.9	**340.4**	347.4	--
TM4	108.56	6.12	386.9	**371.6**	--	--
TM5	116.47	6.57	360.6	**345.3**	--	**343.2**
TM6	112.75	6.36	372.5	**365.1**	368.2	--
CVM1	0.00	7.14	331.5	**319.9**	--	320.4

Table S18. Adjusted ethanol dose and weight changes in male rats

Subject	ivermectin dose (mg/kg)	Ethanol dose (gr/kg)	Baseline Weight (g)	D3 Weight (g)	D5 Weight (g)	D7 Weight (g)
			**210-364**			
TF1	141.78	11.19	211.6	213	--	--
TF2	132.51	10.46	226.4	**223.6**	--	224.4
TF3	126.58	9.99	237	**234.1**	238.7	--
TF4	95.98	10.82	218.8	**216.8**	--	--
TF5	83.60	9.43	251.2	**242.6**	--	246.5
TF6	88.09	9.93	238.4	244.8	--	--
CVF1	0.00	9.49	249.5	**248.5**	--	255.6

Table S19. Adjusted ethanol dose and weight changes in female rats

Subject	Weight (g)	ivermectin dose (mg/kg)	Ethanol dose (g/kg)	Time of FBC	HB (g/dl)	HTC (%)	RBC ($x10^6$/ul)	MCV (fl)	MCH (pg)	MCHC (g/dl)	WBC ($x10^3$/ul)	PLT ($x10^3$/ul)
					9.0-16.2	37.2-48.3	6.7-8.4	51-58.6	12.2-21.3	22.4-36.6	5.6-13.7	487-1291
TM1	303.9	98.72	7.79	72 hours	15.1	45.5	8.1	56.2	18.6	33.2	9.6	**377.0**
TM2	333.2	90.04	7.11	168 hours	13.3	40.0	7.0	57.1	19.0	33.3	11.1	848
TM3	346.9	86.48	6.83	140 hours	14.8	44.5	8.1	55.0	18.3	33.3	12.6	821
TM4	386.9	108.56	6.12	72 hours	12.3	38.6	6.9	55.8	17.8	31.9	**14.3**	946
TM5	360.6	116.47	6.57	168 hours	14.9	45.2	7.8	57.8	19.1	33.0	7.9	1082
TM6	372.5	112.75	6.36	140 hours	13.7	40.6	7.5	53.8	18.2	33.7	9.4	919
CVM1	331.5	0.00	7.14	168 hours	15.1	44.3	7.8	56.9	19.4	34.1	8.0	1156
All values from the animal provider https://www.envigo.com, FBC: full blood count, Hb: hemoglobin, HTC: hematocrit, RBC: red blood cells, MCV: mean corpuscular volume, MCH: mean cospuscular hemoglobin, MCHC: mean corpuscular hemoglobin concentration, WBC: white blood cells, PLT: platelets												

Table S20. Full blood count of male rats

Subject	Weight (g)	ivermectin dose (mg/kg)	Ethanol dose (g/kg)	time of FBC	HB (g/dl)	HTC (%)	RBC ($x10^6$/ul)	MCV (fl)	MCH (pg)	MCHC (g/dl)	WBC ($x10^3$/ul)	PLT ($x10^3$/ul)
					13.2-15.4	37.9-44.8	7.1-8.4	50.5-56.5	17.9-20.2	33.2-37.7	2.6-15.2	206-1447
TF1	211.6	141.78	11.19	72 hours	**11.5**	37.9	**6.06**	**62.5**	19	**30.3**	10.2	1043
TF2	226.4	132.51	10.46	168 hours	13.7	**34**	**5.45**	**62.4**	**25.1**	**40.3**	2.7	412
TF3	237	126.58	9.99	140 hours	**12.7**	38.8	**6.55**	**59.2**	19.4	**32.7**	10.1	1299
TF4	218.8	95.98	10.82	72 hours	**11.5**	**36.7**	**6.11**	**60.1**	18.8	**31.3**	7.1	956
TF5	251.2	83.60	9.43	168 hours	13.9	41	**7.02**	**58.4**	19.8	33.9	8.1	1317
TF6	238.4	88.09	9.93	140 hours	**13.1**	38.8	**6.49**	**60**	20.2	33.8	4.9	1009
CVF1	249.5	0.00	9.49	168 hours	*	*	*	*	*	*	*	*

All values from the animal provider https://www.envigo.com, FBC: full blood count, Hb: hemoglobin, HTC: hematocrit, RBC: red blood cells, MCV: mean corpuscular volume, MCH: mean cospuscular hemoglobin, MCHC: mean corpuscular hemoglobin concentration, WBC: white blood cells, PLT: platelets
*clotted sample

Table S21. Full blood count of female rats

Subject	Baseline Weight (g)	Absolute lung weight (g)*	Relative lung weight (%)*	Absolute liver weight (g)	Relative liver weight (%)
	**321-617**	**1.33-2.61**	**0.31-0.67**	**9.3-18.7**	**2.4-3.9**
TM1	**303.9**	1.72	0.57%	10.61	3.5%
TM2	333.2	1.58	0.47%	11.93	3.6%
TM3	346.9	1.51	0.43%	13.15	3.8%
TM4	386.9	1.87	0.48%	13.32	3.4%
TM5	360.6	1.88	0.52%	10.48	2.9%
TM6	372.5	1.45	0.39%	13.59	3.6%
CVM1	331.5	1.84	0.55%	11.06	3.3%

All values from https://www.envigo.com/ except those marked* taken from Piao et al. J Toxicol Pathol (2013); 26: 29-34

Table S22. Absolute and relative weights of lungs and livers of male rats

Subject	Baseline Weight (g)	Absolute lung weight (g)*	Relative lung weight (%)*	Absolute liver weight (g)	Relative liver weight (%)
	**210-364**	**0.9-2.0**	**0.35-0.83**	**6.0-11.6**	**2.5-4.1**
TF1	211.6	1.35	0.64%	7.65	3.61%
TF2	226.4	1.50	0.66%	8.96	3.96%
TF3	237	1.33	0.56%	8.16	3.44%
TF4	218.8	1.50	0.69%	7.41	3.39%
TF5	251.2	1.72	0.69%	8.85	3.52%
TF6	238.4	1.49	0.63%	7.14	3.00%
CVF1	249.5	1.54	0.62%	10.15	4.07%

All values from https://www.envigo.com/ except those marked* taken from Piao et al. J Toxicol Pathol (2013); 26: 29-34

Table S23. Absolute and relative weights of lungs and livers of female rats
